# Methamphetamine and HIV-1 Tat Synergistically Induce Microglial Pyroptosis Via Activation of the AIM2 Inflammasome

**DOI:** 10.1007/s10753-025-02266-9

**Published:** 2025-02-19

**Authors:** Lin Miao, Haowei Wang, Xue Yang, Lisha Xu, Ruike Xu, Hanxin Teng, Yue Zhang, Yingjie Zhao, Genmeng Yang, Xiaofeng Zeng

**Affiliations:** 1 NHC Key Laboratory of Drug Addiction Medicine, School of Forensic Medicine, Kunming Medical University, Kunming, China; 2 Department of Pathogen Biology and Immunology, School of Basic Medical Science, Kunming Medical University, Kunming, China

**Keywords:** Methamphetamine, HIV-1 Tat, AIM2, Pyroptosis, Microglia, DsDNA

## Abstract

**Objective:**

Human immunodeficiency virus (HIV)-infected individuals who abuse methamphetamine (METH) exhibit more severe neurotoxicity and cognitive impairment. Pyroptosis, a programmed cell death pathway mediated by the inflammasome, has been implicated in various neurological diseases. This study aimed to elucidate the role of the AIM2 inflammasome in METH- and HIV-1 Tat-induced pyroptosis in human brain tissue and in vitro models.

**Methods:**

Postmortem brain tissue from HIV-infected individuals with a history of METH abuse was analyzed for pyroptosis markers and AIM2 inflammasome components using immunohistochemistry, immunofluorescence, and Western blotting. BV2 microglial cells were lentivirally transduced to knockdown AIM2 expression. DNA damage was assessed using Western blotting and the comet assay. Expression of pyroptosis-related proteins was evaluated by electron microscopy, Western blotting, and immunofluorescence. Cell viability was measured using the CCK8 assay.

**Results:**

Elevated levels of pyroptosis markers and AIM2 inflammasome components were observed in brain tissue from HIV-infected METH users. METH and Tat synergistically induced pyroptosis in BV2 cells in a time- and concentration-dependent manner, accompanied by DNA damage and activation of the AIM2 inflammasome. Knockdown of AIM2 significantly reduced the expression of pyroptosis-related proteins.

**Conclusion:**

METH and HIV-1 Tat proteins synergistically induce microglial pyroptosis by activating the AIM2 inflammasome through dsDNA damage. These findings suggest that targeting the AIM2 inflammasome may be a promising therapeutic strategy for HIV-associated neurocognitive disorder (HAND).

**Graphical abstract:**

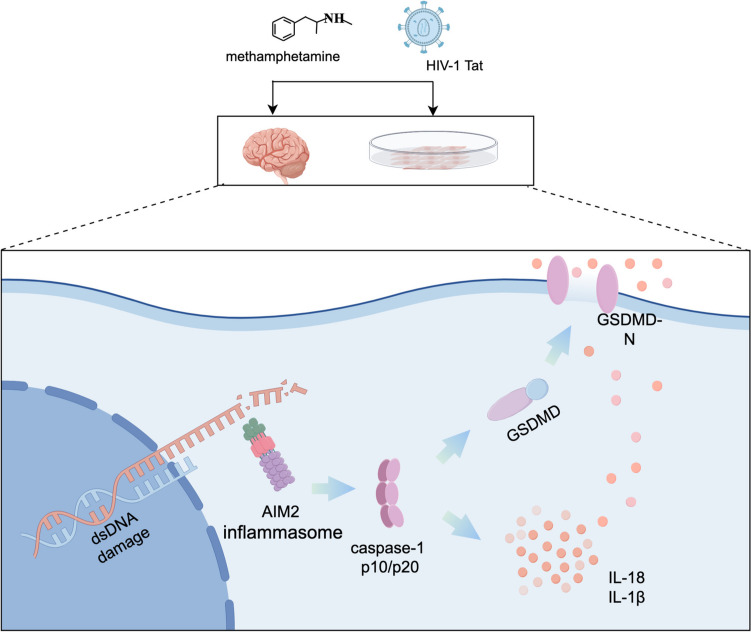

**Supplementary Information:**

The online version contains supplementary material available at 10.1007/s10753-025-02266-9.

## Introduction

Methamphetamine (METH) abuse has become a serious public health problem worldwide, and the incidence of syringe sharing and high-risk sexual behavior among METH abusers greatly increases the risk of spreading and infecting the human immunodeficiency virus (HIV) [1, 2]. HIV-1 Tat protein is a key regulatory protein encoded by HIV-1 and plays an important role in HIV-1 infection [3–6]. Current evidence suggests that METH and HIV-1 Tat protein can enhance each other and synergistically induce the generation of neurotoxicity [7–9], but the underlying mechanism has not been fully elucidated.

Pyroptosis, a distinct mode of inflammatory cell death from apoptosis, is often triggered by external pathogenic factors such as viruses or bacteria. This process frequently involves the release of a substantial quantity of inflammatory mediators [10, 11]. The classical pyroptosis pathway is mediated by the assembly of the activated inflammasome [12, 13]. Upon inflammasome assembly, Caspase-1 is activated, leading to the cleavage and aggregation of its p10 and p20 fragments [14, 15]. Activated Caspase-1 cleaves GSDMD, generating the biologically active GSDMD-N fragment. GSDMD-N perforates the cell membrane, resulting in cell swelling and rupture [16]. Concurrently, Caspase-1 cleaves the precursors of IL-1β and IL-18 into their mature forms, which are subsequently released through pores formed by GSDMD-N, influencing surrounding cells [17–19]. Studies have demonstrated elevated expression of *GSDMD*, a key gene for pyroptosis, in a METH-induced rat model [20]. Additionally, pyroptosis has been implicated in HIV-induced neurotoxicity through various mechanisms [18, 21]. However, the specific mechanisms underlying pyroptosis in the synergistic neurotoxicity induced by METH and HIV-1 Tat protein remain to be fully elucidated.

The Absent in Melanoma 2 (AIM2) is a type of DNA receptor whose C-terminal HIN-200 domain can recognize exogenous and endogenous double-stranded DNA (dsDNA) [22]. Upon recognition of various damaged dsDNAs in vivo and ex vivo, AIM2 initiates the binding of its PYD structural domain to the PYD structural domain on the ASC, which in turn leads to the assembly of the inflammasome by the binding of the CARD on the ASC to the CARD structural domain on the pro-caspase-1. This ultimately results in the activation of Caspase-1 and the induction of pyroptosis [23]. However, the role of the AIM2 inflammasome in the synergistic induction of pyroptosis by METH and HIV-1 Tat proteins remains unclear.

Herein, we identified elevated pyroptosis and expression of AIM2 inflammasome-associated proteins in the brain tissue of HIV-infected methamphetamine abusers. In vitro experiments further confirmed that methamphetamine and HIV-1 Tat proteins can synergistically damage dsDNA, leading to the activation of the AIM2 inflammasome and subsequent pyroptosis. Lentiviral knockdown of the *AIM2* gene in BV2 cells demonstrated that microglial pyroptosis induced by methamphetamine and HIV-1 Tat proteins is primarily mediated through dsDNA damage-induced AIM2 inflammasome activation. This study aimed to elucidate the specific mechanism by which methamphetamine and HIV-1 Tat proteins synergistically induce pyroptosis using human brain tissue samples and in vitro experiments. Our findings provide a potential avenue for developing novel therapeutic strategies for methamphetamine-abusing patients with HIV-associated neurocognitive disorder (HAND).

## Results

### Elevated Expression Levels of Pyroptosis-related Proteins in the Prefrontal Cortex of HIV-infected METH Users

**Fig. 1:**
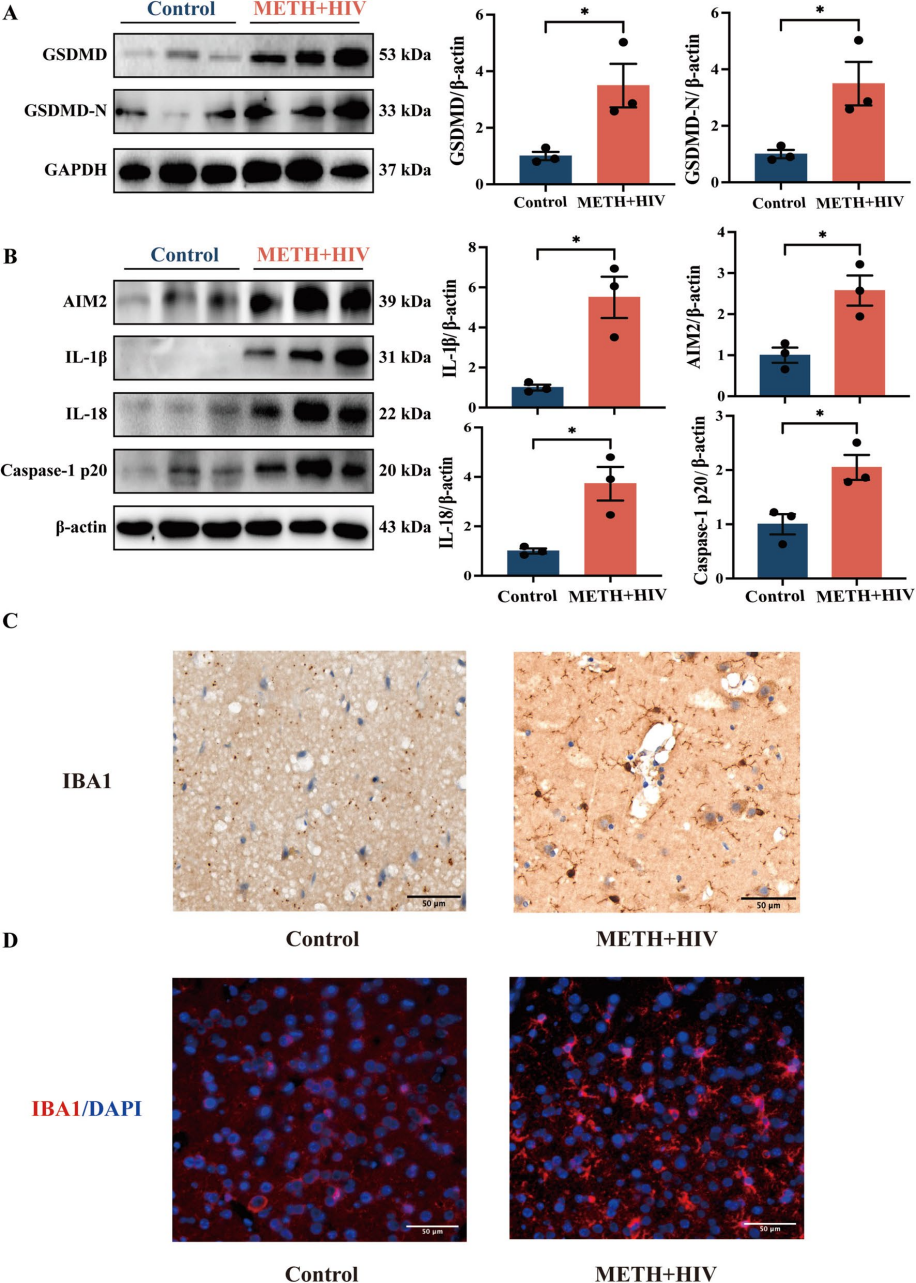
Elevated expression of pyroptosis-related proteins in the prefrontal cortex of the brain of HIV-1-infected METH abusers. (**A**) WB analysis of GSDMD and GSDMD-N protein expression in the prefrontal cortex of both groups. (**B**) The expression of AIM2, IL-1β, IL-18, and Caspase-1 p20 proteins in the prefrontal cortex. (**C**) IBA1 expression in IHC-stained the prefrontal cortex (scale bars, 50 µm). (**D**) IBA1 expression in IF-stained the prefrontal cortex (scale bars, 50 µm). The data are presented as mean ± SEM. n ≥ 3 per group (3 different individuals). *: *p* < 0.05, compared to the respective control group. All experiments were performed in triplicate at a minimum

We collected the prefrontal cortex from the brains of deceased HIV-infected methamphetamine abusers from the Kunming Medical University Judicial Identification Centre. Compared to normal populations, we found elevated levels of GSDMD and GSDMD-N proteins in brain tissue of HIV-infected METH abusers (Fig. 1A). Furthermore, an examination of AIM2 Inflammasome-associated proteins revealed overexpression of AIM2, IL-1β, IL-18, and Caspase-1 p20 in METH abusers infected with HIV compared to the control group (Fig. 1B). Ionized calcium binding adapter molecule 1 (IBA1) is a specific marker protein in microglia [7]. In the present study, microglia activation was detected using immunofluorescence (IF) and immunohistochemistry (IHC) techniques. The results demonstrated a significant increase in area and number of iba1 + cells in the METH + HIV group compared to the control group (Fig. 1C, D; Supplementary Fig. 2A, B). These findings suggest that HIV-infected METH users exhibited elevated expression levels of pyroptosis and AIM2 Inflammasome-associated proteins in the prefrontal cortex, accompanied by microglia activation.

### METH and HIV-1 Tat Protein Synergistically Induced Pyroptosis in BV2 Cells

**Fig. 2:**
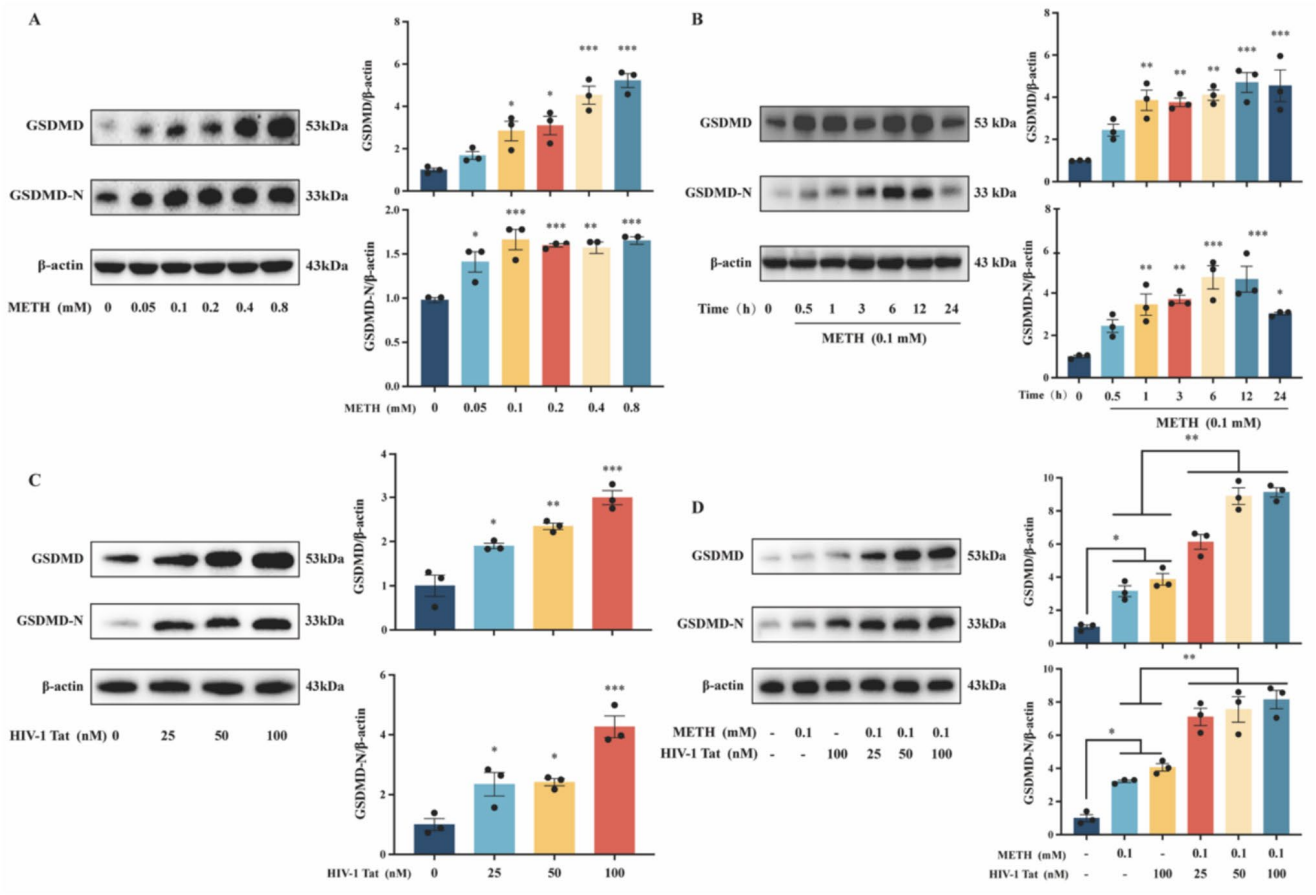
Time- and dose-dependent induction of pyroptosis in BV2 cells by METH and HIV-1 Tat protein. (**A**) The expression of proteins GSDMD and GSDMD-N after the action of different concentrations of METH in BV2 cells. (**B**) The expression of GSDMD and GSDMD-N proteins after different times of METH action on BV2 cells. (**C**) The expression of GSDMD and GSDMD-N proteins after the action of different concentrations of HIV-1 Tat protein in BV2 cells. (**D**) The expression of GSDMD and GSDMD-N proteins after METH and HIV-1 Tat protein synergistic action on BV2 cells. The data are presented as mean ± SEM. n = 3 per group (3 different culture batches). *: *p* < 0.05, **: *p* < 0.01, ***: *p* < 0.001, compared to the respective control group. All experiments were performed in triplicate at a minimum

To further elucidate the exact mechanism of pyroptosis and AIM2 inflammasome activation in the brain tissue of HIV-infected METH users, we conducted in vitro experiments using BV2 cells as the study subjects. To successfully model the synergistic induction of microglial pyroptosis by METH and HIV-1 Tat protein in vitro, we first applied different concentrations of METH (0, 0.05, 0.1, 0.2, 0.4, 0.8 mM) (Fig. 2A) and HIV-1 Tat protein (0, 25, 50, 100 nM) (Fig. 2C) to BV2 cells for various durations (0, 0.5, 1, 3, 6, 12, 24 h) (Fig. 2B). The optimal time and concentration points for METH and HIV-1 Tat proteins' effects on BV2 cells were determined by assaying the expression of pyroptosis-related proteins. The N-terminal GSDMD (GSDMD-N) has been identified as the primary executor of pyroptosis. Based on the changes in GSDMD-N protein expression, we determined that 0.1 mM of METH and 100 nM of HIV-1 Tat protein acting on BV2 cells for 12 h were the most optimal concentration and time points. Moreover, this combination was also found to be the optimal delivery concentration for synergistically inducing pyroptosis (Fig. 2D).

**Fig. 3:**
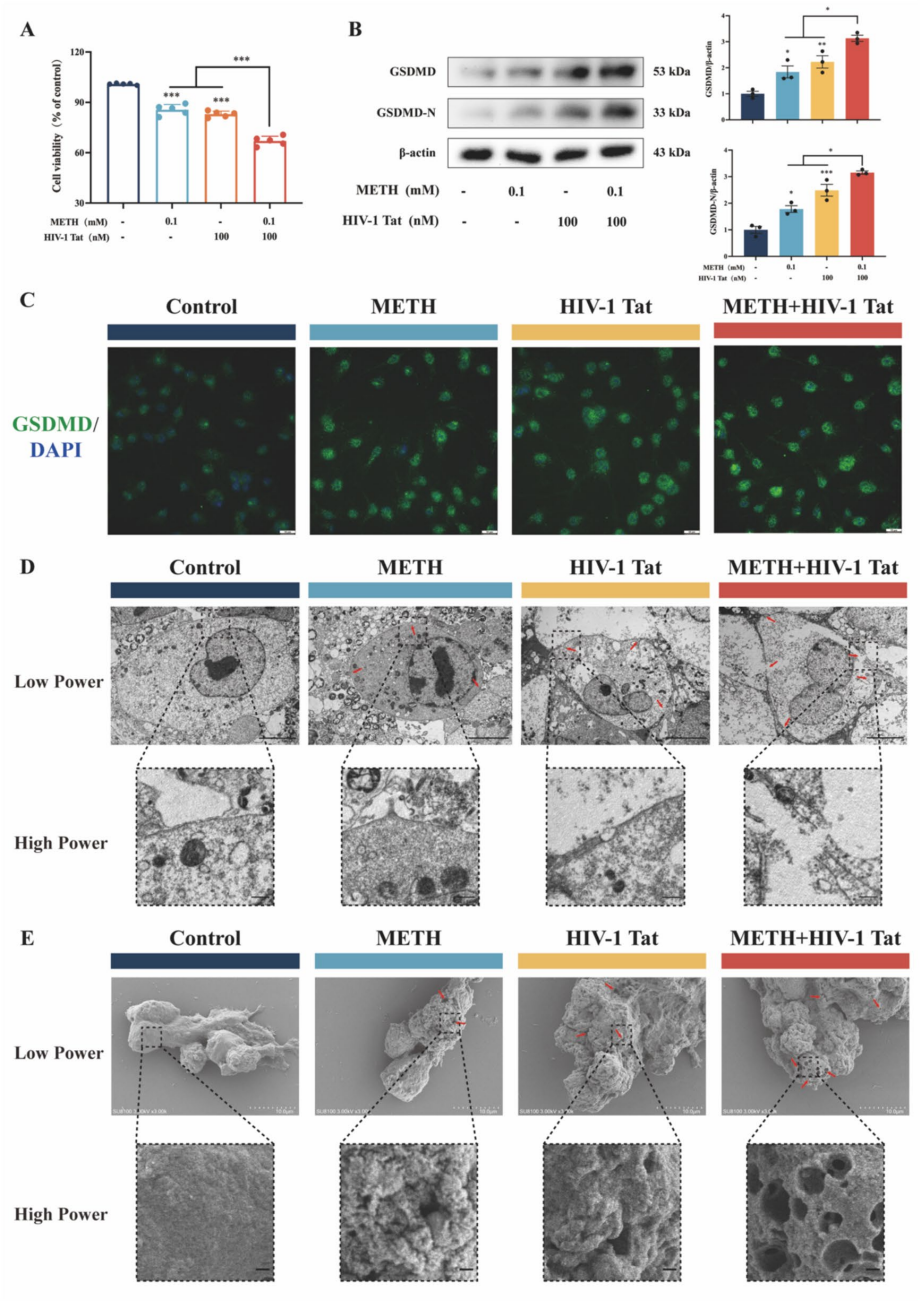
METH and HIV-1 Tat protein synergistically induced pyroptosis in BV2 cells. (**A**) The CCK8 assay analyses the cellular activity of BV2 cells in four groups. (**B**) WB analysis of GSDMD and GSDMD-N protein expression among the four groups. (**C**) The typical image of IF of GSDMD proteins in BV2 cells (scale bars, 10 µm). (**D**) The typical image of TEM of BV2 cell (scale bars, low power 5 µm and high power 500 nm). (**E**) The typical image of SEM of BV2 cell (scale bars, low power 10 µm and high power 500 nm). The data are presented as mean ± SEM. n ≥ 3 per group (3 different culture batches). **: *p* < 0.01, ***: *p* < 0.001, compared to the respective control group. All experiments were performed in triplicate at a minimum

After treating BV2 cells with METH and HIV-1 Tat proteins at the previously determined time and dose, changes in cell activity were detected using the CCK8 kit. We found that cell activity was significantly reduced in the METH and HIV-1 Tat protein groups compared to the control group. This reduction in cell activity was even more pronounced when the two proteins worked synergistically (Fig. 3A). Western blot analysis revealed a significant increase in the expression of GSDMD and GSDMD-N in the METH and HIV groups compared to the control group, and this increase was further exacerbated in the METH + HIV-1 Tat protein group (Fig. 3B). These findings were corroborated by immunofluorescence staining (Fig. 3C; Supplementary Fig. 2C). Furthermore, using scanning electron microscopy (SEM) and transmission electron microscopy (TEM), we observed that the BV2 cell membranes were disrupted by the action of METH and HIV-1 Tat proteins, with the formation of pores on the cell surface. This phenomenon was more pronounced when METH and HIV-1 Tat protein acted together (Fig. 3D, E). The aforementioned results collectively suggest that METH and HIV-1 Tat protein synergistically induce pyroptosis in BV2 cells.

### METH and HIV-1 Tat Proteins Synergistically Damage BV2 Cell DsDNA and Activate AIM2 Inflammasome

**Fig. 4:**
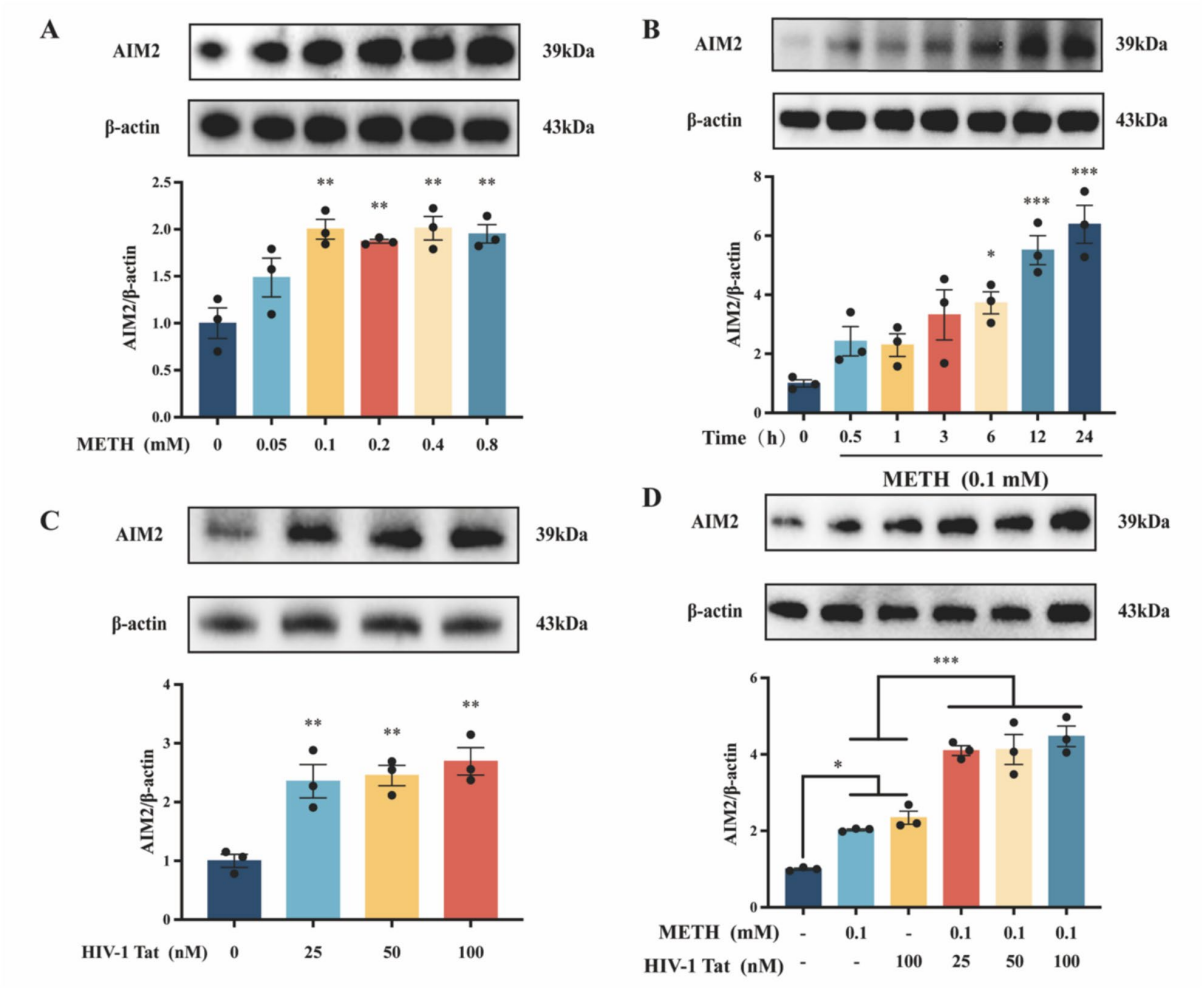
Time- and dose-dependent induction of AIM2 protein activation by METH and HIV-1 Tat proteins in BV2 cells. (**A**) The expression of protein AIM2 after the action of different concentrations of METH in BV2 cells. (**B**) The expression of AIM2 protein after different times of METH action on BV2 cells. (**C**) The expression of protein AIM2 after the action of different concentrations of HIV-1 Tat protein in BV2 cells. (**D**) The expression of AIM2 protein after METH and HIV-1 Tat protein synergistic action on BV2 cells. The data are presented as mean ± SEM. n ≥ 3 per group (3 different culture batches). *: *p* < 0.05, **:* p* < 0.01, ***: *p* < 0.001, compared to the respective control group. All experiments were performed in triplicate at a minimum

Similarly, we treated BV2 cells with various concentrations of METH (0, 0.05, 0.1, 0.2, 0.4, and 0.8 mM) (Fig. 4A) and HIV-1 Tat proteins (0, 25, 50, and 100 nM) (Fig. 4C) for different durations (0, 0.5, 1, 3, 6, 12, and 24 h) (Fig. 4B). By examining the protein expression of AIM2 in BV2 cells, we reconfirmed that a concentration of 0.1 mM METH and 100 nM HIV-1 Tat protein acting for 12 h was the optimal combination for inducing AIM2 activation. This concentration was also identified as the optimal administration concentration for synergistic activation of AIM2 by METH and HIV-1 Tat protein (Fig. 4D), consistent with our previous findings.

**Fig. 5:**
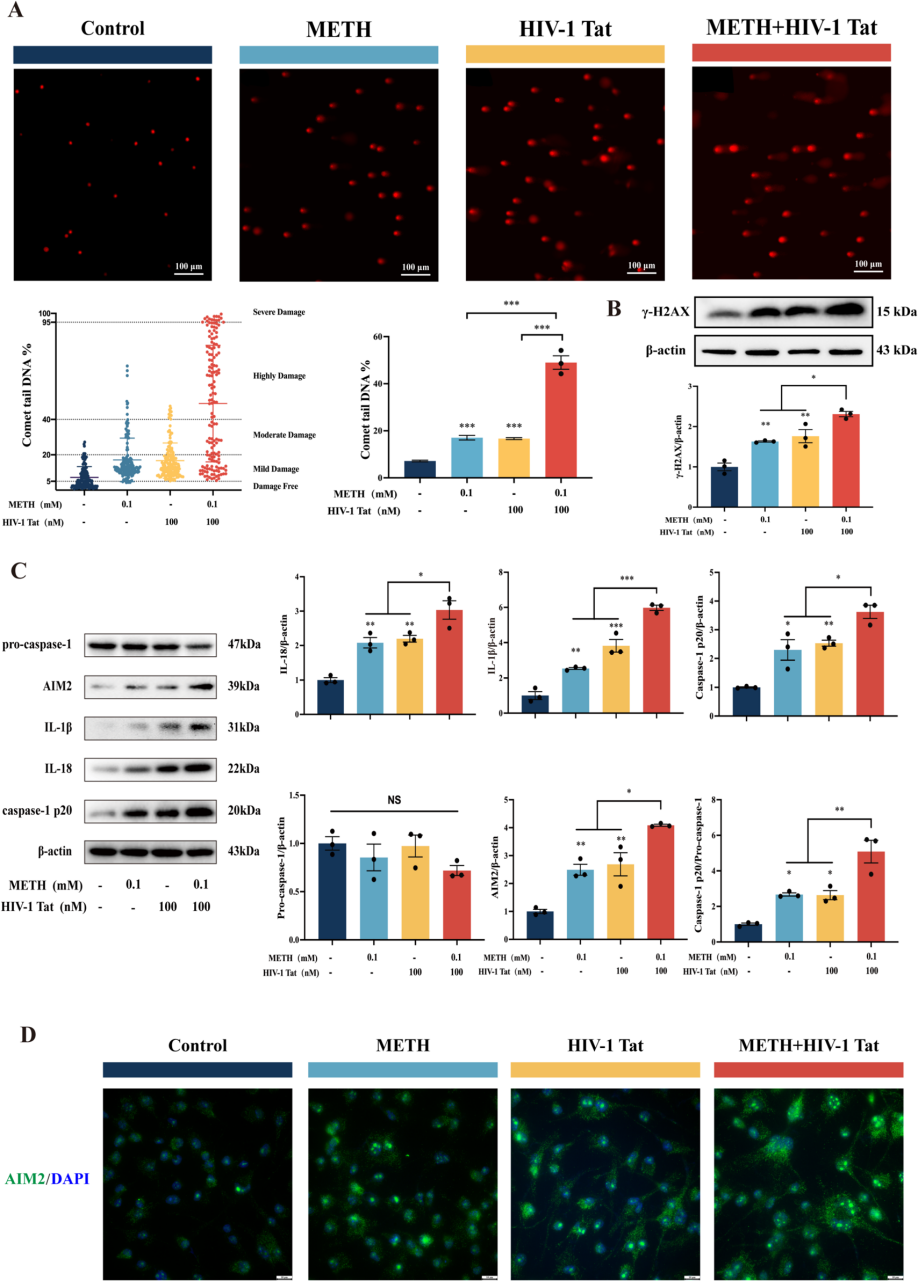
METH and HIV-1 Tat proteins synergistically damage BV2 cell dsDNA and activate AIM2 Inflammasome. (**A**) The comet assay analyses dsDNA damage in four groups of BV2 cells. (**B**) WB analysis of γ-H2AX protein expression among the four groups. (**C**) The expression of proteins pro-caspase-1, AIM2, IL-1β, IL-18, and Caspase-1 p20 in BV2 cells. (**D**) The typical image of IF of AIM2 protein in BV2 cells (scale bars, 10 µm). The data are presented as mean ± SEM. n ≥ 3 per group (3 different culture batches). **: *p* < 0.01, ***: *p* < 0.001, compared to the respective control group. All experiments were performed in triplicate at a minimum

The AIM2 inflammasome is capable of recognizing damaged dsDNA and subsequent activation [24]. To assess dsDNA damage in BV2 cells, we employed the comet assay. Our findings revealed a significant increase in dsDNA damage in the METH and HIV-1 Tat protein groups compared to the control group, with a particular emphasis on mild damage. Notably, the METH + HIV-1 Tat protein group exhibited substantial dsDNA damage, primarily reaching high and severe levels (Fig. 5A). These results were further corroborated by Western blot detection of γ-H2AX protein expression (Fig. 5B). It is well-established that upon activation of the AIM2 inflammasome, pro-caspase-1 is cleaved into its active fragment, p20, and p10. We next conducted Western blot analysis to examine the protein expression of pro-caspase-1, AIM2, IL-1β, IL-18, and Caspase-1 p20. Compared to the control group, the expression of AIM2 inflammasome-associated proteins was significantly elevated in both the METH and HIV-1 Tat protein groups, with even higher levels observed in the METH + HIV-1 Tat protein groups (Fig. 5C). These results were also validated by immunofluorescence staining (Fig. 5D; Supplementary Fig. 2D). Collectively, these findings suggest that METH and HIV-1 Tat proteins can synergistically damage dsDNA in BV2 cells and activate the AIM2 inflammasome.

### Effects of AIM2 Inflammasome Inhibition On Synergistic Induction of Pyroptosis in BV2 Cells By METH and HIV-1 Tat Protein

To elucidate the role of the AIM2 inflammasome in the synergistic induction of pyroptosis in BV2 cells by METH and HIV-1 Tat proteins, we employed a lentiviral vector expressing a short hairpin RNA (shRNA) targeting AIM2.

Initially, we established BV2 cell models stably transfected with either a control vector or the AIM2 lentivirus. The lentiviruses carried the GFP gene, allowing for the identification of AIM2 knockout cells by their green fluorescence. Fluorescence microscopy was used to assess the transfection efficiency of BV2 cells with various AIM2 shRNA sequences at different multiplicities of infection (MOIs) (Supplementary Fig. 1A). We found that fluorescence expression was highest in BV2 cells transfected with vectors and AIM2 lentiviruses at an MOI of 4 without affecting cellular viability. Consequently, an MOI of 4 was determined as the optimal for transfection with both the control vector and different AIM2 shRNA sequences. Following transfection with vectors and AIM2 lentiviruses, we collected BV2 cells and performed Western blot (WB) and real-time quantitative polymerase chain reaction (RT-qPCR) experiments to evaluate the knockdown efficiency of the different AIM2 shRNA sequences (Supplementary Fig. 1B, C). The results demonstrated that the shRNA-1 group exhibited the lowest levels of AIM2 protein and mRNA expression compared to the vector-transfected cells. Therefore, shRNA-1 was selected as the optimal sequence for AIM2 lentiviral transfection. To determine the optimal concentration of puromycin for selecting stably transfected BV2 cells, we treated non-transfected cells with varying concentrations of puromycin for 72 h and assessed cell viability using the CCK8 kit (Supplementary Fig. 1D). We found that a puromycin concentration of 2 μg/mL effectively killed most BV2 cells, making it suitable for subsequent screening of stably transfected cells. Finally, we collected BV2 cells transfected with vectors and AIM2 lentiviruses under the optimized conditions and examined the effect of stable transfection using WB and fluorescence microscopy (Supplementary Fig. 1E, F). The results revealed a significant reduction in AIM2 protein expression in the shRNA-1 group compared to the vector group. Furthermore, fluorescence microscopy showed that the fluorescence expression of cells in the shRNA-NC and shRNA-1 groups closely resembled that of cells in the bright field, indicating successful construction of BV2 cell models stably transfected with either the control vector or the AIM2 lentivirus, which could be utilized for further experiments.

**Fig. 6:**
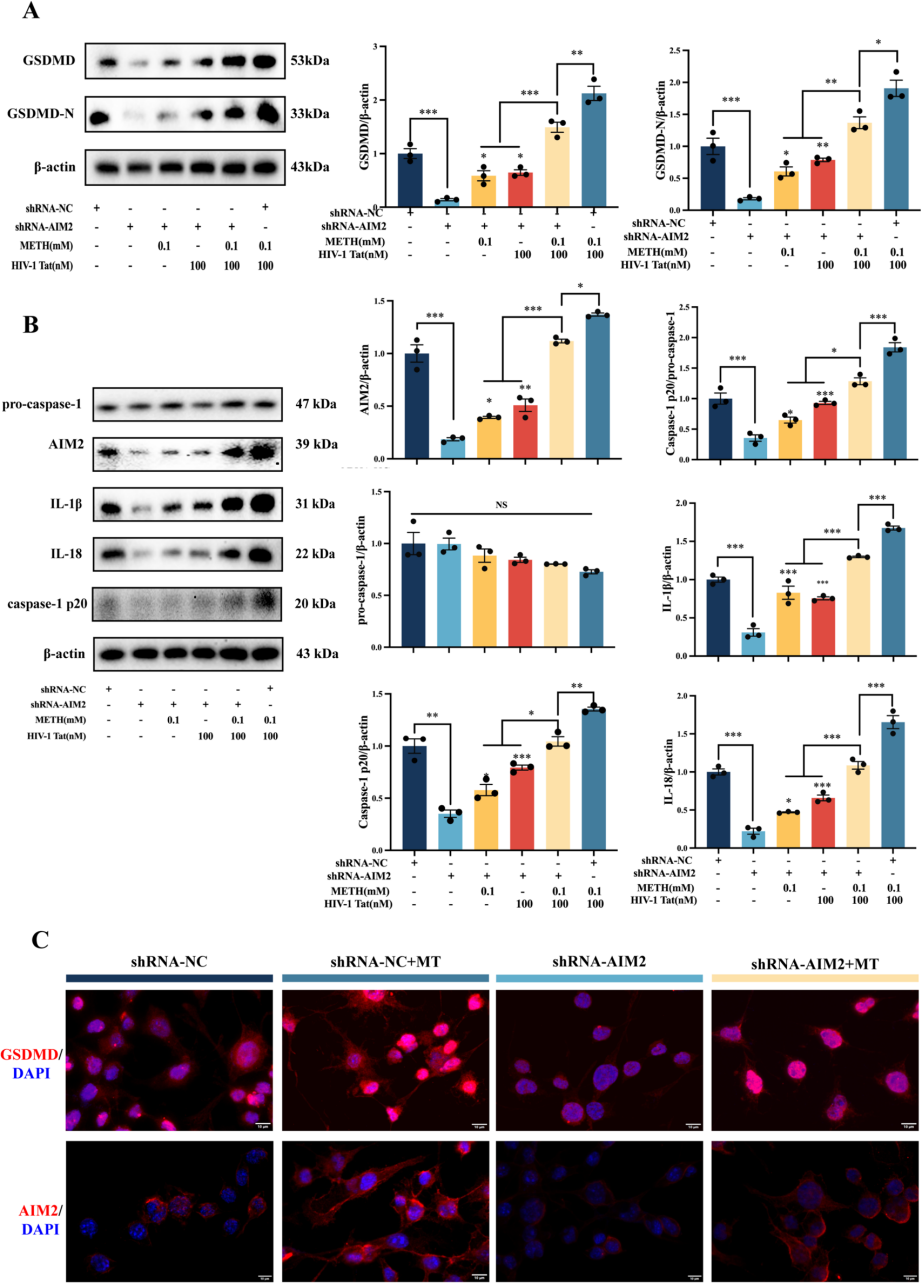
Effects of AIM2 Inflammasome inhibition on synergistic induction of pyroptosis in BV2 cells by METH and HIV-1 Tat protein. (**A**) WB analysis of GSDMD and GSDMD-N protein expression among the six groups. (**B**) The expression of proteins pro-caspase-1, AIM2, IL-1β, IL-18, and Caspase-1 p20 in BV2 cells stably transfected with vector or AIM2 lentivirus. (**C**) The typical image of IF of GSDMD and AIM2 proteins in BV2 cells stably transfected with vector or AIM2 lentivirus (scale bars, 10 µm). The data are presented as mean ± SEM. n ≥ 3 per group (3 different culture batches). **: *p* < 0.01, ***: *p* < 0.001, compared to the respective control group. All experiments were performed in triplicate at a minimum

To investigate the role of the AIM2 inflammasome in the synergistic induction of pyroptosis by METH and HIV-1 Tat proteins in BV2 cells, we firstly assessed changes in cell viability using the CCK8 kit. The results showed that increased cell viability in the METH + HIV-1 Tat + shRNA-1 group compared to the METH + HIV-1 Tat + shRNA-NC group (Supplementary Fig. 2E). Meanwhile, we examined changes in the expression of cell pyroptosis-associated proteins using WB analysis in BV2 cells stably transfected with vectors and AIM2 lentivirus (Fig. 6A). The results demonstrated a significant reduction in the protein expression levels of GSDMD and GSDMD-N in the METH + HIV-1 Tat + shRNA-1 group compared to the METH + HIV-1 Tat + shRNA-NC group. Similarly, WB analysis revealed a significant decrease in the expression of AIM2 inflammasome-associated proteins in the METH + HIV-1 Tat + shRNA-1 group compared to the METH + HIV-1 Tat + shRNA-NC group (Fig. 6B). These findings were further confirmed by immunofluorescence staining (Fig. 6C; Supplementary Fig. 2F, G). Collectively, these results suggest that METH and HIV-1 Tat proteins synergistically induce pyroptosis in BV2 cells through the activation of the AIM2 inflammasome.

## Discussion

The mechanisms underlying neurotoxicity and addiction induced by METH, the most widely abused amphetamine, have been extensively studied. However, the mechanisms of neurotoxicity resulting from its combination with HIV are even more complex [1, 9, 25–28]. Pyroptosis, a newly identified mode of programmed cell death closely linked to inflammasome activation, has also been implicated in neurological damage [11–13, 29]. In brain tissues of HIV-infected individuals who abused METH, we detected elevated expression of GSDMD and GSDMD-N, suggesting that METH and HIV may induce pyroptosis in human brain tissue cells. Microglia, the primary line of defense against toxic injury in the central nervous system, are highly sensitive to neurotoxic effects and neuronal damage [30, 31]. Neuroinflammation, resulting from the dysregulation of immune cells in the central nervous system, is closely associated with inflammasome activation, of which microglia are key drivers [32, 33]. Furthermore, pyroptosis is a type of programmed cell death that is intimately connected to inflammatory responses [18, 31, 34]. In addition, we observed elevated expression of IBA1 in the prefrontal cortex of HIV-infected METH abusers. Therefore, BV2 cells were employed as a study subject for in vitro experiments to elucidate the mechanisms by which METH and HIV-1 Tat proteins synergistically induce pyroptosis.

While elevated expression of GSDMD and GSDMD-N has been the primary focus in studies on pyroptosis, recent research has also demonstrated that caspase-3 activation can induce pyroptosis characterized by elevated GSDME expression [35–37]. However, there is a paucity of studies investigating the induction of pyroptosis by METH and HIV-1 Tat proteins. Previous findings have shown that METH induces cell death in HT22 cells primarily through the expression of GSDME rather than GSDMD [38]. However, pore formation on the membrane of HT22 cells was not observed in that study and was not classified as pyroptosis. The activation of the inflammasome, a process upstream of pyroptosis, is often accompanied by the release of GSDMD-N, which recognizes phospholipid molecules in the cell membrane and forms pores, a hallmark characteristic of pyroptosis [39–41]. In our study, the combined action of METH and HIV-1 Tat protein on BV2 cells resulted in cell death predominantly characterized by elevated GSDMD expression. Furthermore, scanning electron microscopy and transmission electron microscopy revealed that both METH and HIV-1 Tat proteins could lead to the formation of pores in the cell membrane compared to the control group, exhibiting a synergistic effect when acting together. Therefore, our results suggest that METH and HIV-1 Tat proteins can synergistically induce pyroptosis in microglia.

Inflammasome activation can trigger the innate immune response through the secretion of inflammatory factors and the induction of pyroptosis [40, 42, 43]. Previous studies have identified the activation of NLRP3 and NLRP1 inflammasomes in the hippocampal brain regions of individuals who died from chronic METH exposure [20, 26, 44]. Additionally, significant upregulation of AIM2 and NLRP3 inflammasomes has been observed in HIV-infected macrophages in the presence of cocaine [45]. While the role of NLRP3 inflammasome in METH- and HIV-1-induced pyroptosis is well-established, the involvement of AIM2 inflammasome in the synergistic induction of pyroptosis by METH and HIV-1 remains unclear. Our findings demonstrate that METH and HIV-1 Tat proteins can synergistically induce dsDNA damage in BV2 cells, as evidenced by Western blot, immunofluorescence, and comet assays. Furthermore, we confirmed that METH and HIV-1 Tat proteins can synergistically activate AIM2 inflammasome. Based on these results, we propose that damaged dsDNA is translocated into the cytoplasm to activate the AIM2 inflammasome after METH and HIV-1 Tat proteins synergistically act on microglia. In a previous study, inhibiting NLRP1 expression in rat brains was shown to reduce METH-induced elevated expression of caspase-1, IL-1β, and GSDMD [20]. Similarly, in our study, using lentivirus to knock down the expression of the *AIM2* gene in BV2 cells significantly reduced the level of pyroptosis and increased cell viability. Collectively, these findings suggest that METH and HIV-1 Tat proteins can synergistically induce microglial pyroptosis through dsDNA damage-mediated activation of the AIM2 inflammasome.

In addition, it must be acknowledged that this study has several other limitations. First, it should be noted that gaining access to patient groups who abuse METH and/or are infected with HIV presents significant challenges. Future studies would benefit from obtaining a more diverse range of human samples to achieve a more comprehensive understanding of the issues involved. Secondly, Unlike in vitro cellular assays, the concentrations of HIV-Tat and METH in humans are affected by a number of complex factors. Thus, while our in vitro experiments provide important mechanistic insights, differences in these complex biological concentrations and therapeutic contexts need to be taken into account when applying these findings to the clinic.

In conclusion, our study demonstrates that METH and HIV-1 Tat proteins synergistically induce microglial pyroptosis through dsDNA damage-mediated activation of the AIM2 inflammasome. These findings contribute to the theoretical understanding of the co-induction of neurotoxicity by METH and HIV-1 and offer a potential therapeutic target.

## Materials and Methods

### Cell Culture

The mouse microglial cell line BV2 was obtained from the Chinese Academy of Sciences (Shanghai, China). BV2 cells were cultured in high-glucose Dulbecco's Modified Eagle Medium (DMEM) (VivaCell, Shanghai, China) supplemented with 10% fetal bovine serum (Biological Industries, Israel) and 1% penicillin–streptomycin solution (VivaCell, Shanghai, China). The cell lines were cultured at 37 °C in an incubator with 5% CO_2_ and 95% humidity.

### Human Brain Tissue

All human brain tissue samples were obtained from forensic autopsy cases at the Forensic Appraisal Center of Kunming Medical University. During autopsy, the cause of death was determined through a comprehensive analysis that included macromorphological, histological, toxicological, and biochemical examinations. The prefrontal cortices of collected human brain tissue were utilized for the experiments. Individuals included in the control group should fulfil all three of the following conditions: no history of METH abuse, no HIV infection, and no history of CNS disease. Those who died with a history of both METH abuse and HIV infection were assigned to the experimental group. The history of METH abuse and HIV infection was determined by forensic toxicological analysis and hospital-provided test reports. The experimental protocol was approved by the Medical Ethics Committee of Kunming Medical University (KMMU2022MEC037).

### Reagents

METH was obtained from the National Institute for the Control of Pharmaceutical and Biological Products (Beijing, China). HIV-Tat protein (#HIV-129-c) was purchased from Protein Specialists (Israel).

### Lentiviral Transfection

Three AIM2 shRNA knockdown lentiviral vectors and a lentiviral control vector (VP013-U6-MCS-CMV-ZsGreen-PGK-PURO) were constructed at General Biosystems (Anhui, China). The experiments were conducted following the manufacturer's instructions. To determine optimal conditions for BV2 cell inoculation density and MOI, pre-experimentation infections were performed. Cells were then transfected in the presence of polybrene (General Biosystems, Anhui, China) using the established optimal conditions. Transfection efficiency was verified after 48 h to select the most effective knockdown sequence among the shRNA lentiviral vectors. After 48 h of transfection, cells were further cultured in medium containing puromycin (Solarbio, Beijing, China) to establish a stably transfected cell line.

### Immunohistochemistry Assay

After deparaffinization, hydration, and antigen retrieval of paraffin-embedded brain tissue sections, they were blocked with 10% goat serum (Solarbio, Beijing, China) for 30 min. Each brain tissue sample was incubated overnight with a primary antibody (IBA1, ThermoFisher, dilution ratio: 1:200) at 4 °C. The following day, the PV-9000 DAB detection kit (ZSGB-BIO, Beijing, China) was used for visualization according to the manufacturer's instructions.

### Immunofluorescence Assay

After deparaffinization, hydration, and antigen retrieval, paraffin-embedded brain tissue sections were blocked with 10% goat serum (Solarbio, Beijing, China) for 30 min at room temperature. Subsequently, each brain tissue sample was incubated with a primary antibody (IBA1, #MA5-27,726, ThermoFisher, dilution ratio: 1:200) overnight at 4 °C.

BV2 cells were fixed with 4% paraformaldehyde (Biosharp, Anhui, China) for 30 min, followed by permeabilization with 0.5% Triton X-100 (Solarbio, Beijing, China) at room temperature for 20 min. After blocking the cells with 10% goat serum for 30 min, primary antibodies were incubated at 4 °C overnight. The primary antibodies used included GSDMD (#66,387–1-Ig, Proteintech, dilution ratio: 1:200) and AIM2 (#DF3514, Affinity, dilution ratio: 1:200).

On the second day, the cells were washed with PBS three times and incubated with the corresponding secondary antibody (Invitrogen, USA) at room temperature for 1 h. An anti-quenching sealing agent containing DAPI (Solarbio, Beijing, China) was used for sealing. Fluorescence images were collected using a fluorescence microscope (Leica, Germany).

### Comet Assay

The comet assay was performed following the instructions provided by the reagent kit (Keygen Biotech, Nanjing, China). A three-layer gel was prepared on a slide, and the cell suspension was incorporated into the second layer. The glass slide was placed in a petri dish containing lysis solution for lysis, followed by horizontal electrophoresis. After staining the sample with propidium iodide (PI), the image was observed under a fluorescence microscope and analyzed using Image J software.

### Western Blotting (WB)

Western blotting was performed as described earlier [7]. Briefly, total proteins were extracted from cells and brain tissue, and their concentrations were determined using a BCA detection kit (Beyotime, Shanghai, China). The proteins were incubated with primary antibodies overnight at 4 °C. Subsequently, the samples were incubated with secondary antibodies (Signalway Antibody, USA) at room temperature for 2 h. Protein bands were visualized using ECL chemiluminescence reagent (Biosharp, Anhui, China) and quantitatively analyzed using ImageJ software.

The primary antibodies employed in this study included GSDMD (#66,387–1-Ig, Proteintech, dilution ratio: 1:1000), AIM2 (#66,902–1-Ig, Proteintech, dilution ratio: 1:1000), Caspase-1 p20 (#22,915–1-AP, Proteintech, dilution ratio: 1:1000), Pro-caspase-1 (#ab179515, Abcam, dilution ratio: 1:1000), gamma H2AX (#NB100-2280, Novusbio, dilution ratio: 1:1000), IL-18 (#10,663–1-AP, Proteintech, dilution ratio: 1:1000), and IL-1 beta (#26,048–1-AP, Proteintech, dilution ratio: 1:1000).

### Real-time Quantitative Polymerase Chain Reaction (RT-qPCR)

As described previously [46], total RNA was extracted from BV2 cells using the TransZol Up Plus RNA Kit (TransGen Biotech, China) following the manufacturer's instructions. RNA concentration was subsequently measured using the NanoDrop 2000. RNA samples were then reverse transcribed into cDNA using the Fasting RT Kit (with gDNase) (TIANGEN, China), and relative mRNA expression was quantified using SuperReal PreMix Plus (SYBR Green) (TIANGEN, China).

### Cell Viability Assay

Cell viability was determined using the Cell Counting Kit 8 (Absin, Shanghai, China) following the manufacturer's protocol. The absorbance was measured at 450 nm.

### Statistical Analysis

Statistical analyses were performed using GraphPad Prism version 9.5.0 (GraphPad, USA). To compare the two groups, a Student's t-test was employed. For comparisons involving three or more groups, one-way analysis of variance (ANOVA) followed by Tukey's test was utilized. All experimental results are presented as the mean ± standard error of the mean (SEM), and the significance threshold was set at a *p*-value of less than 0.05.

## Supplementary Information

Below is the link to the electronic supplementary material.

Supplementary file1 (DOCX 689 KB)

Supplementary file2 (DOCX 276 KB)

Supplementary file3 (PDF 27689 KB)

## Data Availability

No datasets were generated or analysed during the current study.
