# Supplementary material for: Methamphetamine and HIV-1 Tat Synergistically Induce Microglial Pyroptosis Via Activation of the AIM2 Inflammasome
Source: Inflammation. 2025 Feb 19;48(5):3300–13. doi: 10.1007/s10753-025-02266-9 (PMC12596323; doi:10.1007/s10753-025-02266-9)
Supplement: Supplementary file 1 — Supplementary file1 (DOCX 689 KB) [file 10753_2025_2266_MOESM1_ESM.docx]

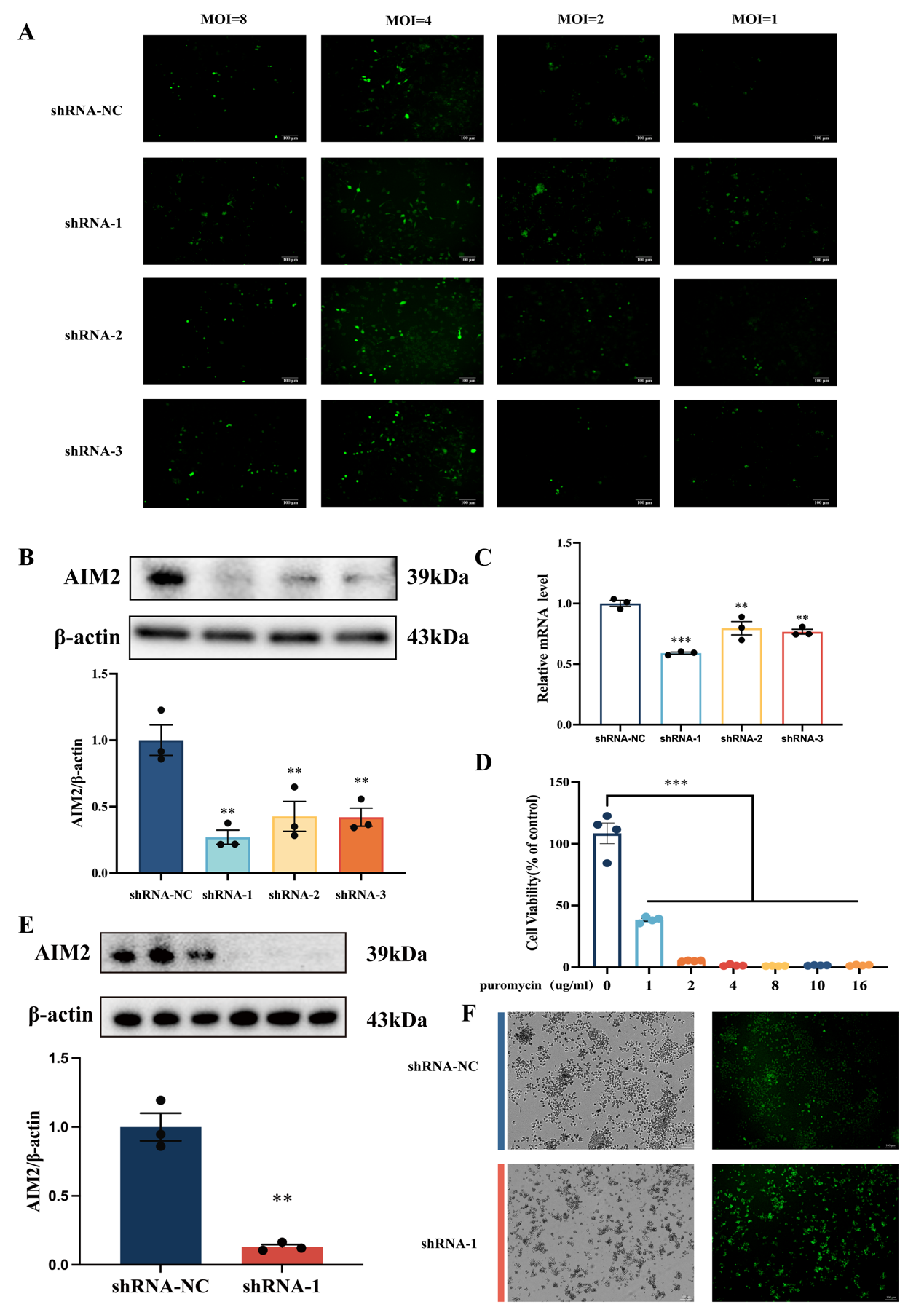


**Supplementary Fig. 1.** The construction of a BV2 cell model stably transfected with vector or AIM2 lentivirus. (A) The representative images of transfection efficiency of BV2 cells with different sequences of vectors or AIM2 lentivirus at different MOI values (scale bars, 100 µm). (B) WB analysis of AIM2 protein expression among different vectors or AIM2 lentivirus sequences. (C) Relative expression of the BV2 cells mRNA AIM2 after transfection of different vectors or AIM2 lentivirus sequences. (D) The CCK8 assay analyzed the effect of different concentrations of puromycin on BV2 cell activity. (E) The expression of protein AIM2 in BV2 cells stably transfected with vector or AIM2 lentivirus. (F) Typical images of stably transfected BV2 cells using vector or AIM2 lentivirus (scale bars, 100 µm). The data are presented as mean ±SEM. n≥ 3 per group (3 different culture batches). **: *p* <0.01, ***: *p* <0.001, compared to the respective control group. All experiments were performed in triplicate at a minimum.
