# Supplementary material for: Methamphetamine and HIV-1 Tat Synergistically Induce Microglial Pyroptosis Via Activation of the AIM2 Inflammasome
Source: Inflammation. 2025 Feb 19;48(5):3300–13. doi: 10.1007/s10753-025-02266-9 (PMC12596323; doi:10.1007/s10753-025-02266-9)
Supplement: Supplementary file 2 — Supplementary file2 (DOCX 276 KB) [file 10753_2025_2266_MOESM2_ESM.docx]

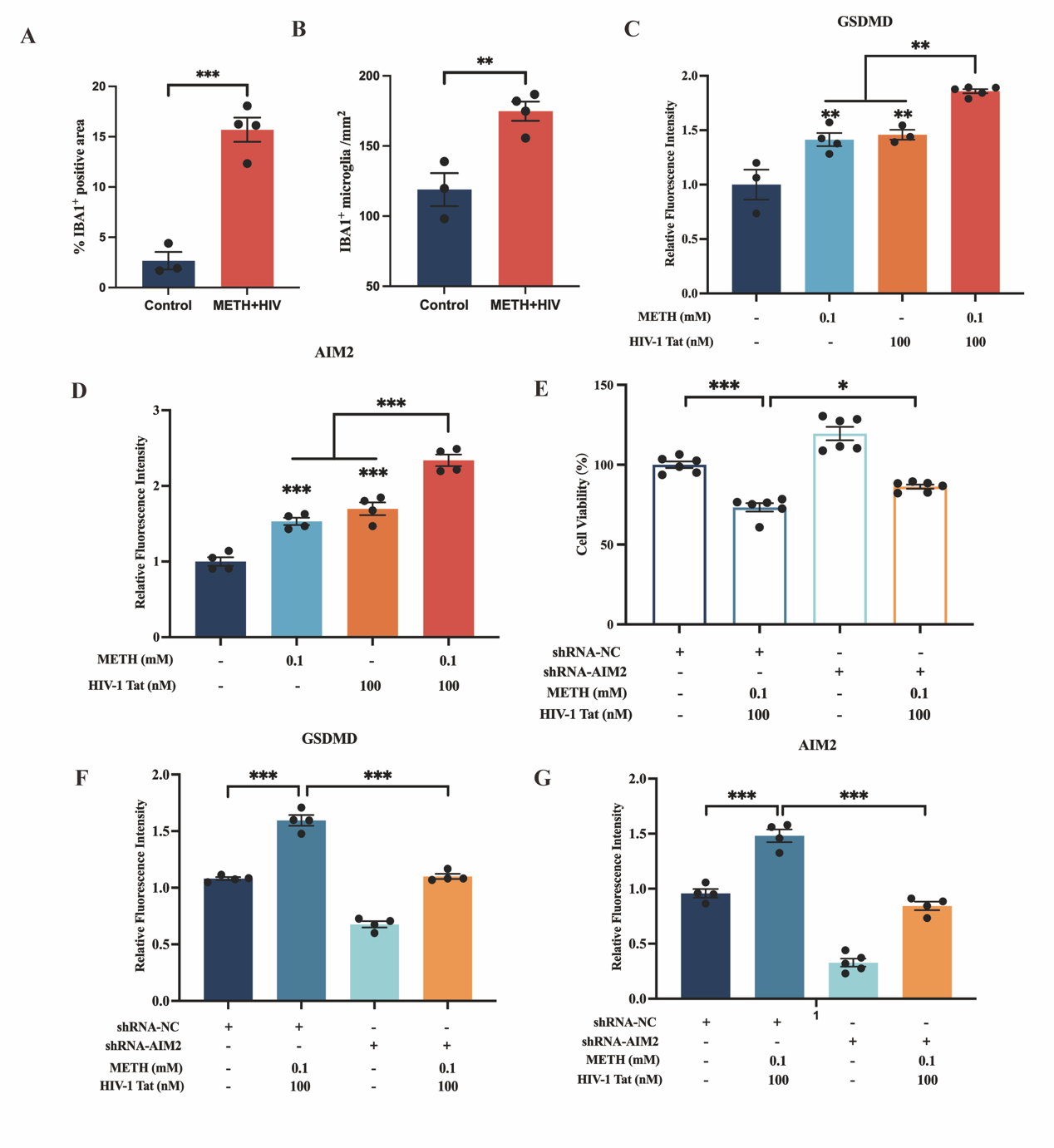


**Supplementary fig. 2.** Statistical results of microscopic data. (A) Quantification and statistical analysis of %IBA1^+^ area. （B）Quantification and statistical analysis of IBA1^+^ microglia/ mm^2^. (C) Quantification and statistical analysis the relative fluorescence intensity of GSDMD. (D) Quantification and statistical analysis the relative fluorescence intensity of AIM2. (E) The CCK8 assay analyses the cellular activity in BV2 cells stably transfected with vector or AIM2 lentivirus. (F) Quantification and statistical analysis the relative fluorescence intensity of GSDMD. (G) Quantification and statistical analysis the relative fluorescence intensity of AIM2. The data are presented as mean ±SEM. n≥3 per group (3 different individuals or culture batches). *: *p* <0.05, **: *p* <0.01, ***: p <0.001, compared to the respective control group. All experiments were performed in triplicate at a minimum.
