# Supplementary material for: Methamphetamine and HIV-1 Tat Synergistically Induce Microglial Pyroptosis Via Activation of the AIM2 Inflammasome
Source: Inflammation. 2025 Feb 19;48(5):3300–13. doi: 10.1007/s10753-025-02266-9 (PMC12596323; doi:10.1007/s10753-025-02266-9)

Uncut Gel Blots

The protein marker was used for this manuscript:
Page Ruler (Thermo Fisher, Cat#: 26616)
Prestained Protein Marker II (Servicebio, Cat#: G2091-250UL)

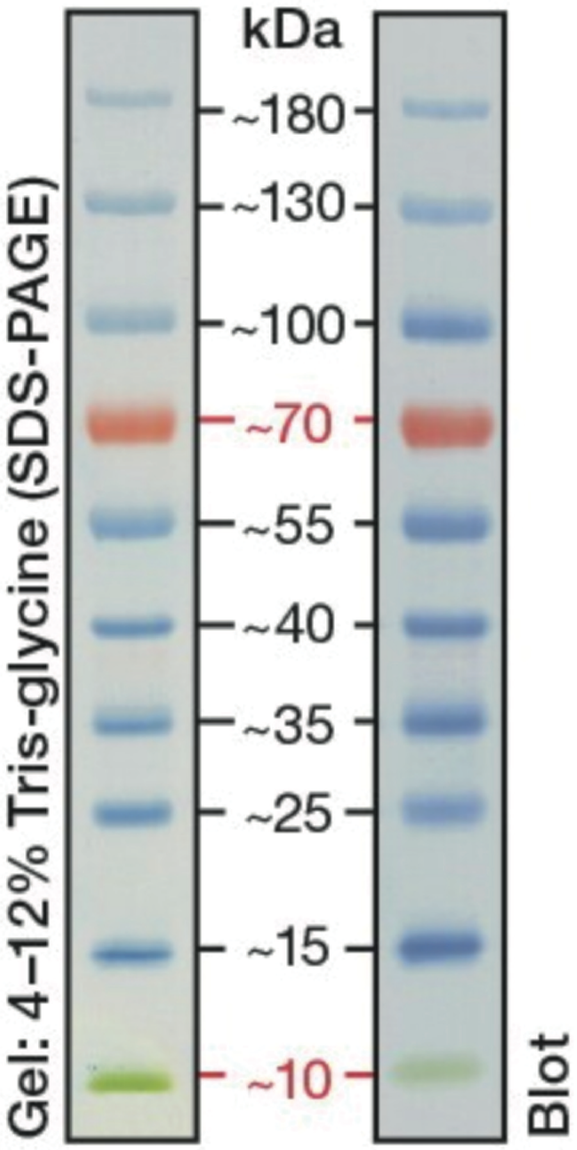

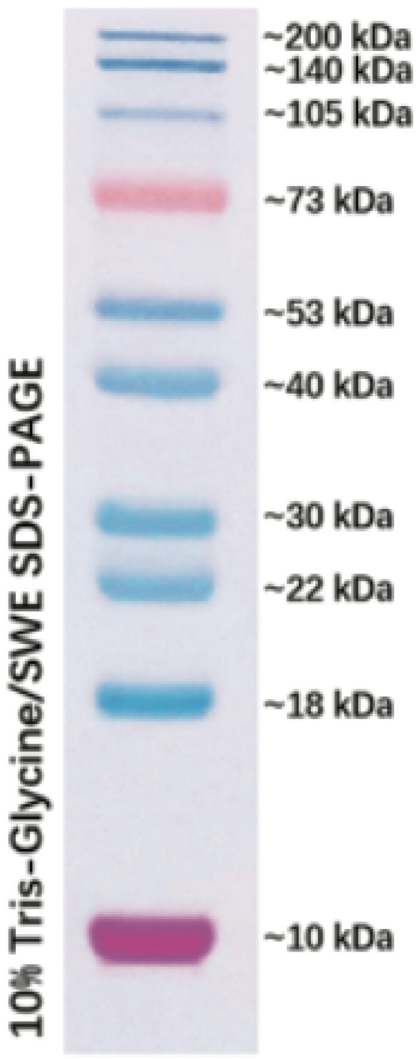

Fig 1. A

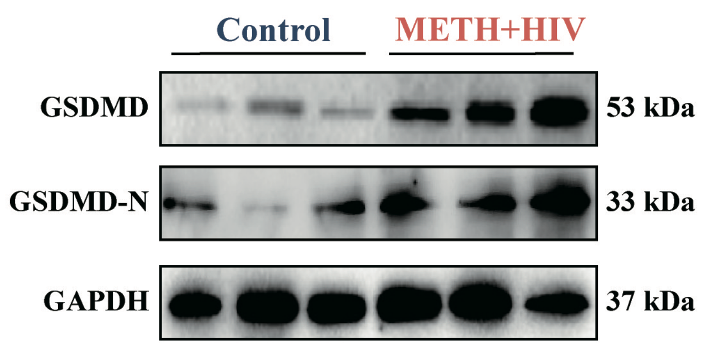

Full unedited gels for Fig 1. A

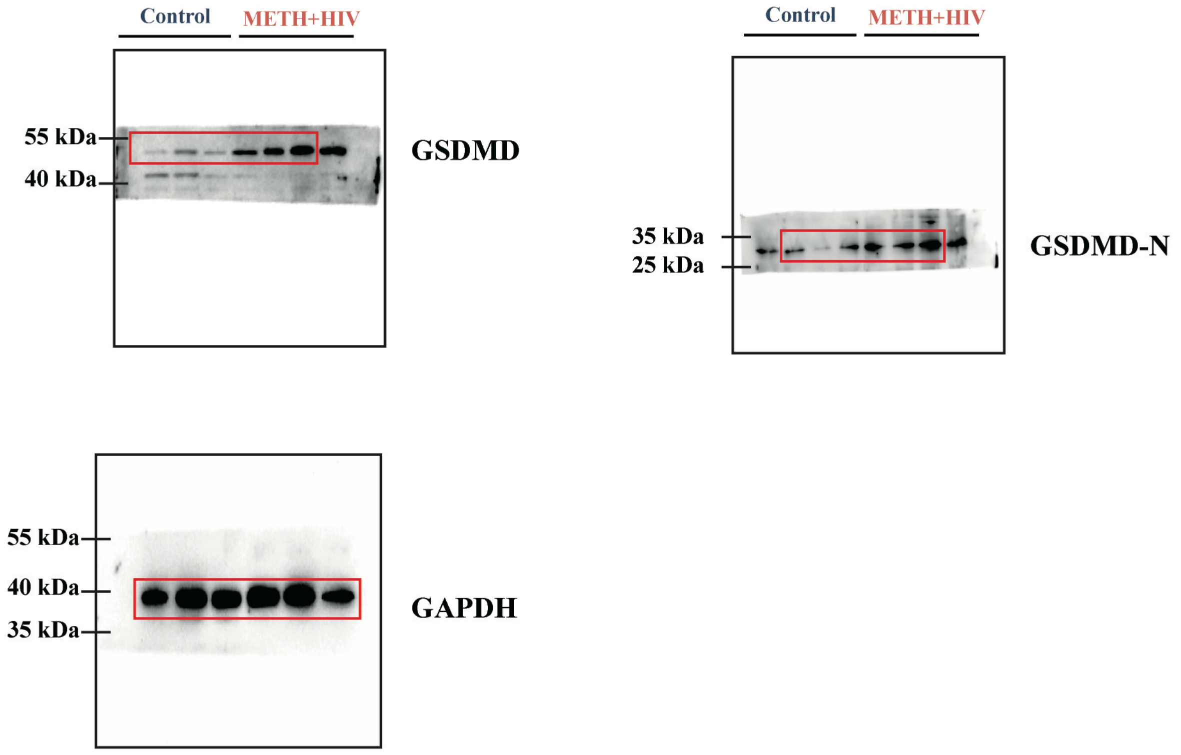

Fig 1. B

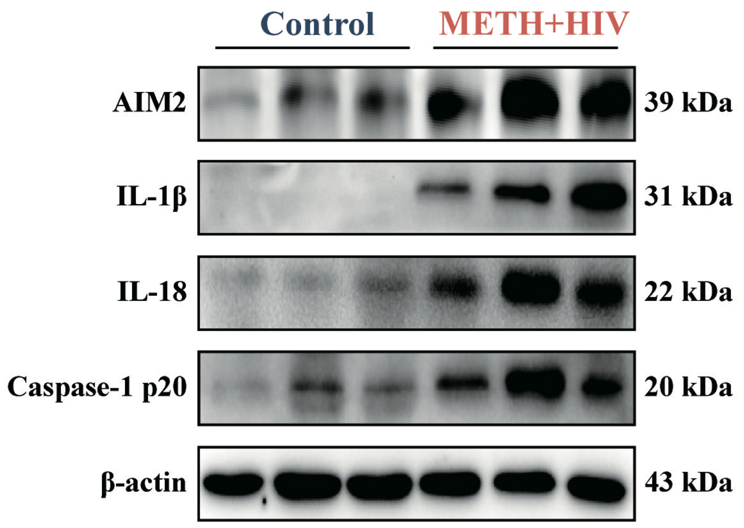

Full unedited gels for Fig 1. B

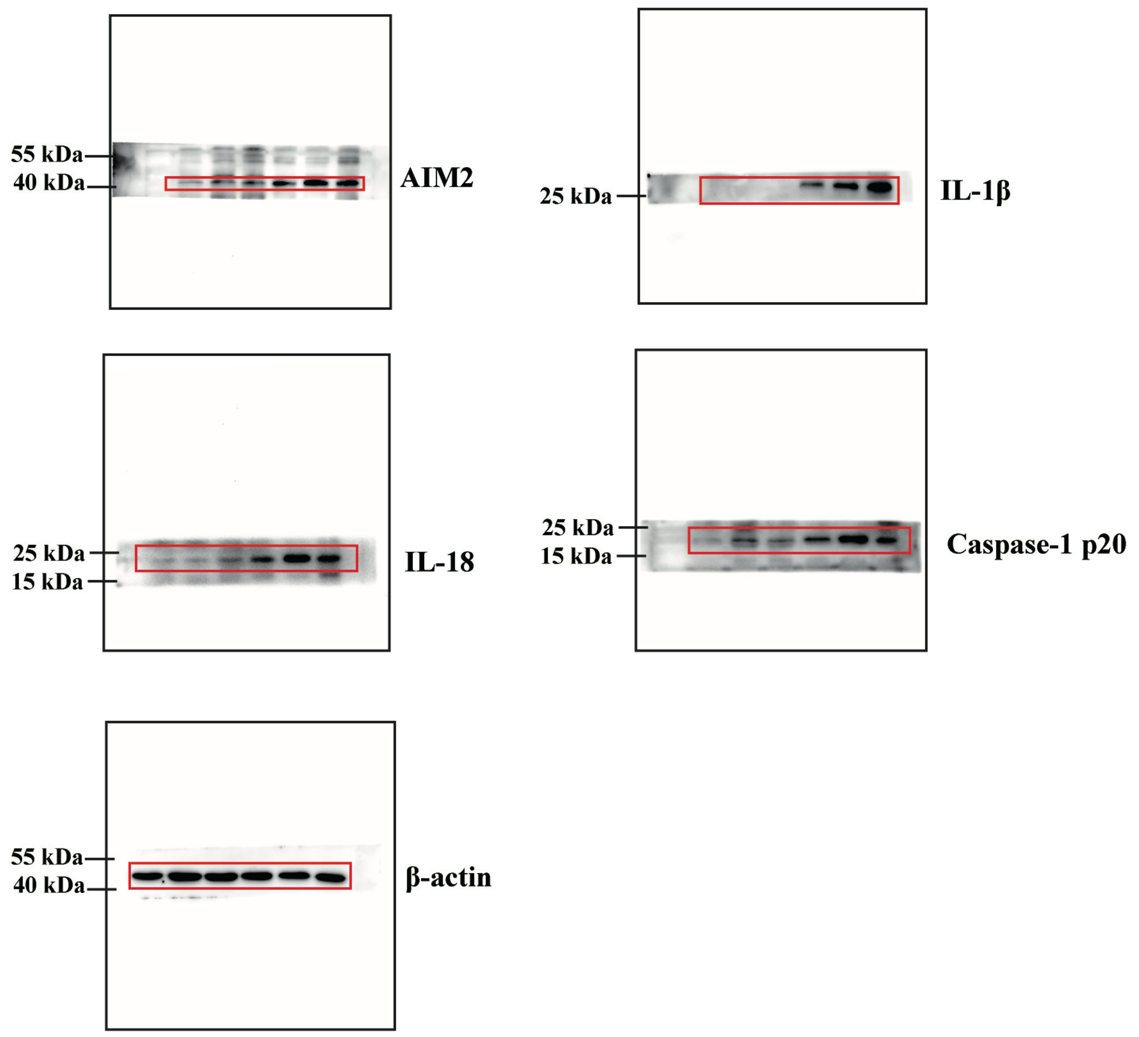

Fig 2. A

Full unedited gels for Fig 2. A

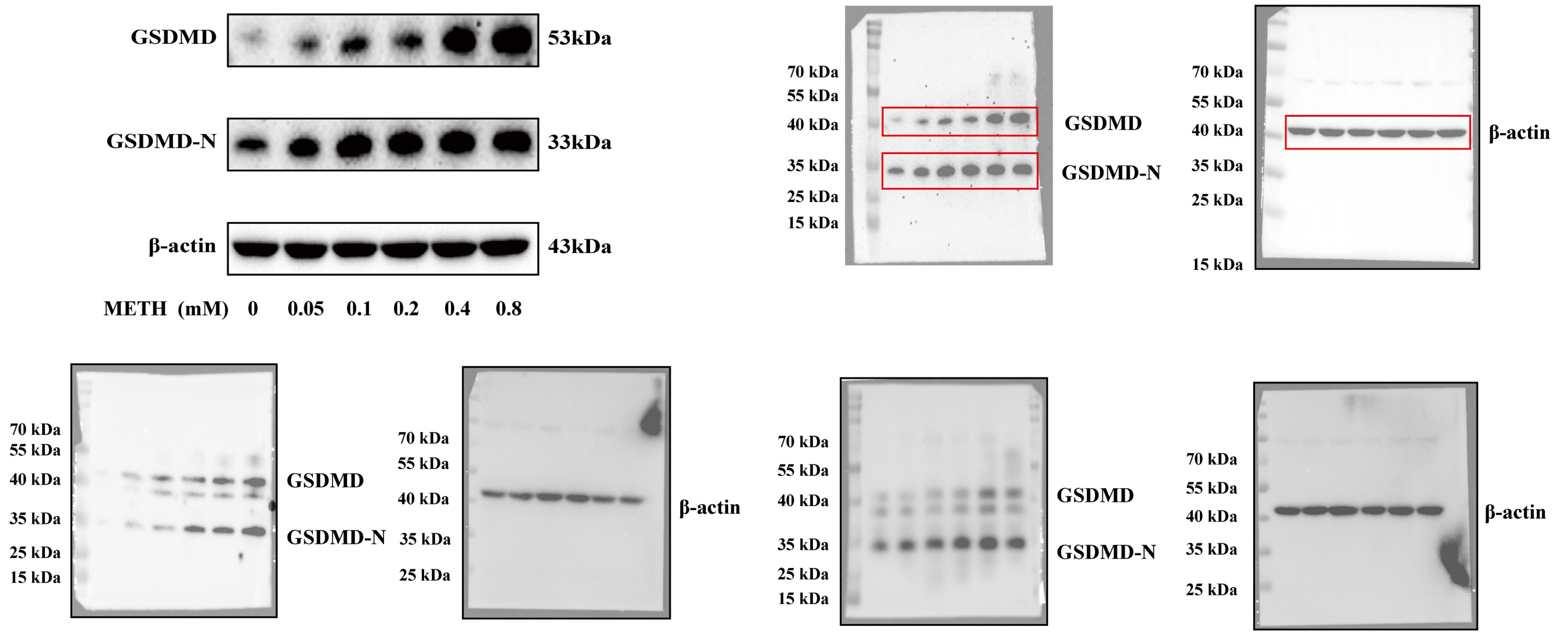

Fig 2. B

Full unedited gels for Fig 2. B

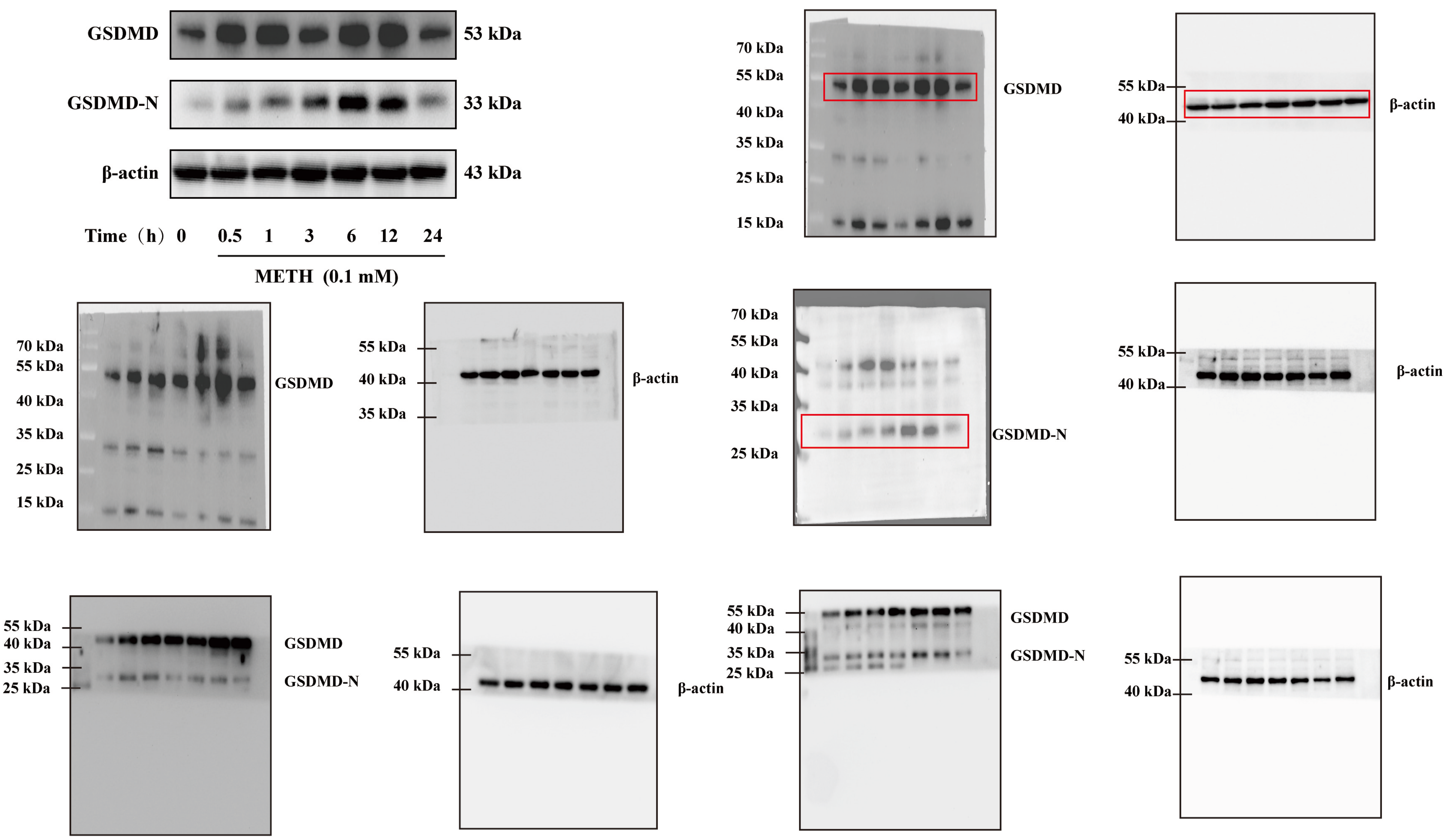

Fig 2. C

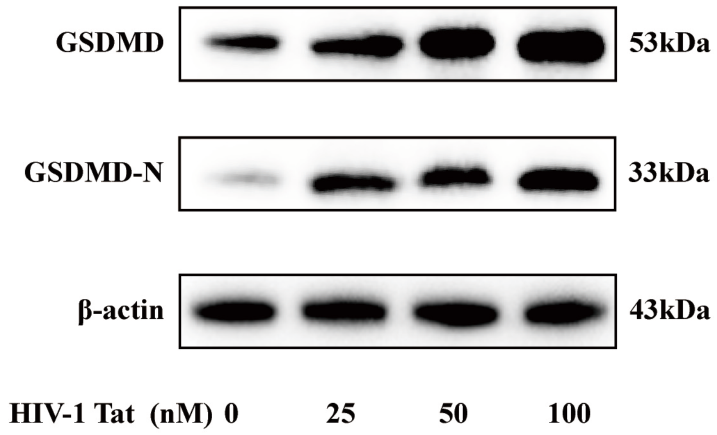

Full unedited gels for Fig 2. C

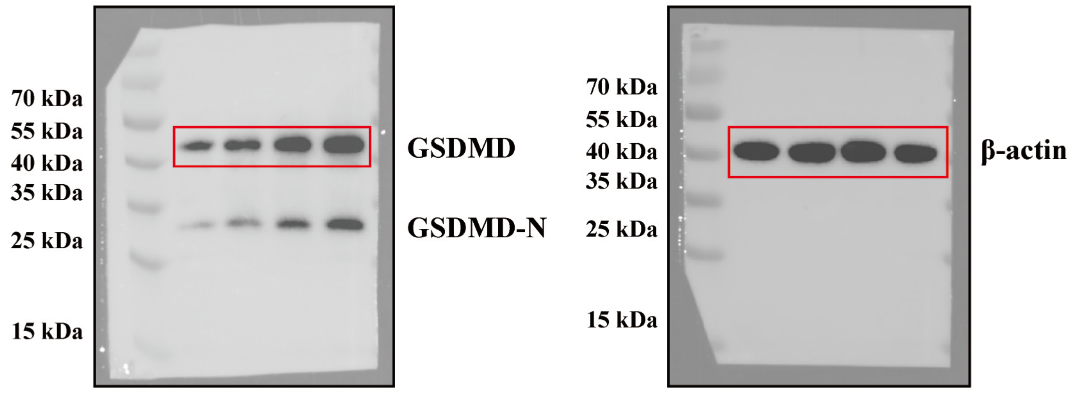

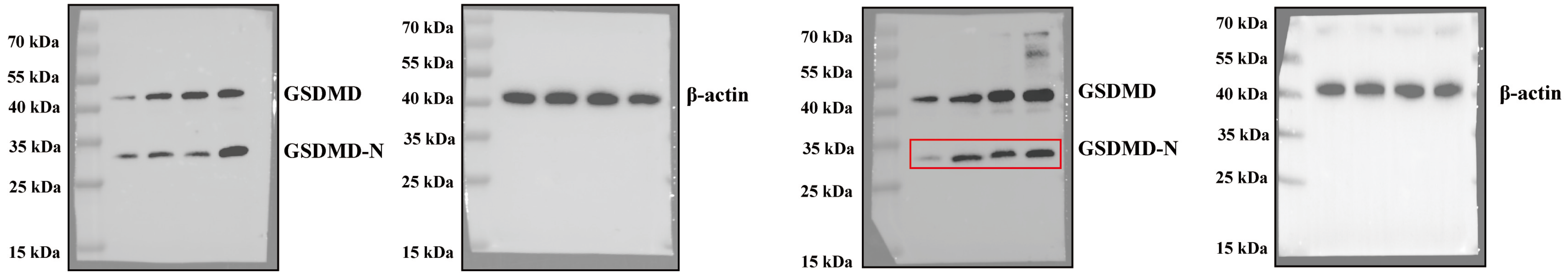

Fig 2. D

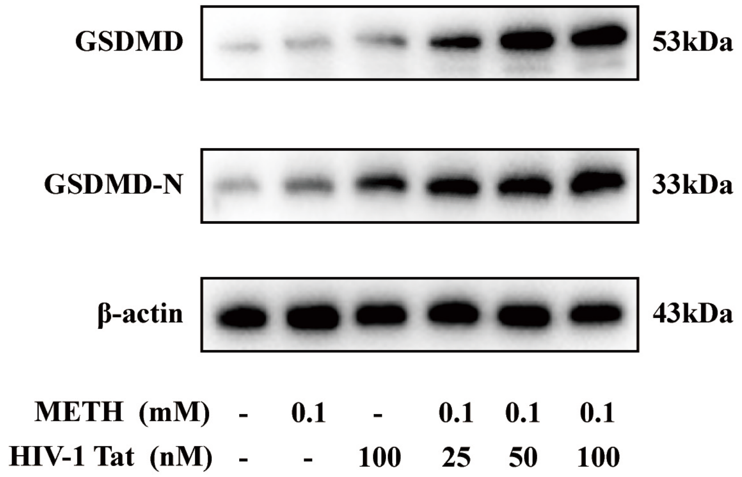

Full unedited gels for Fig 2. D

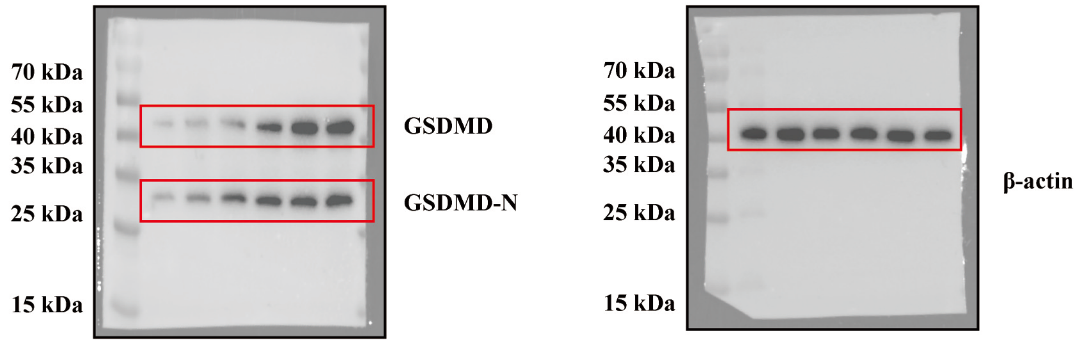

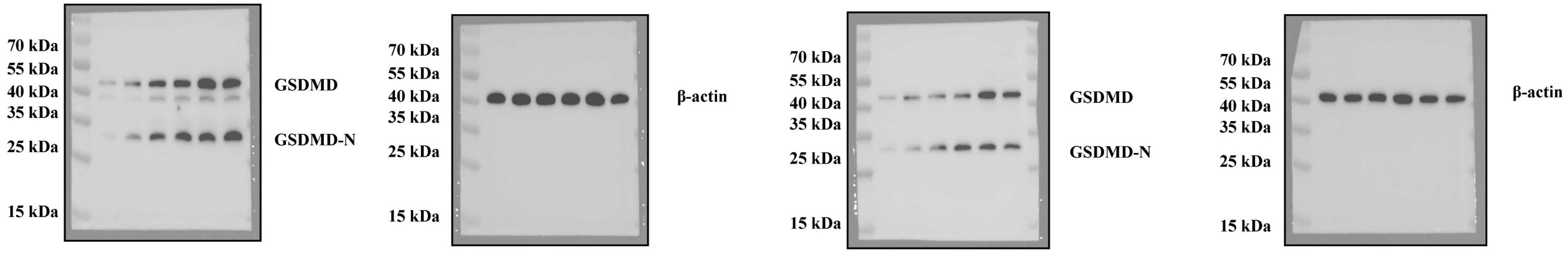

Fig 3.B

Full unedited gels for Fig 3. B

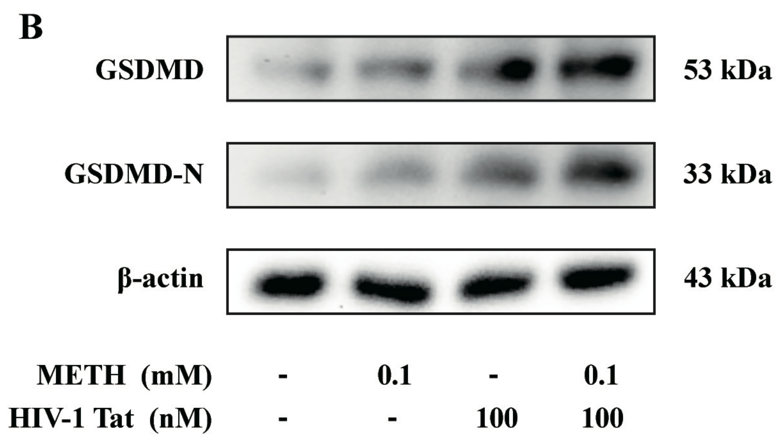

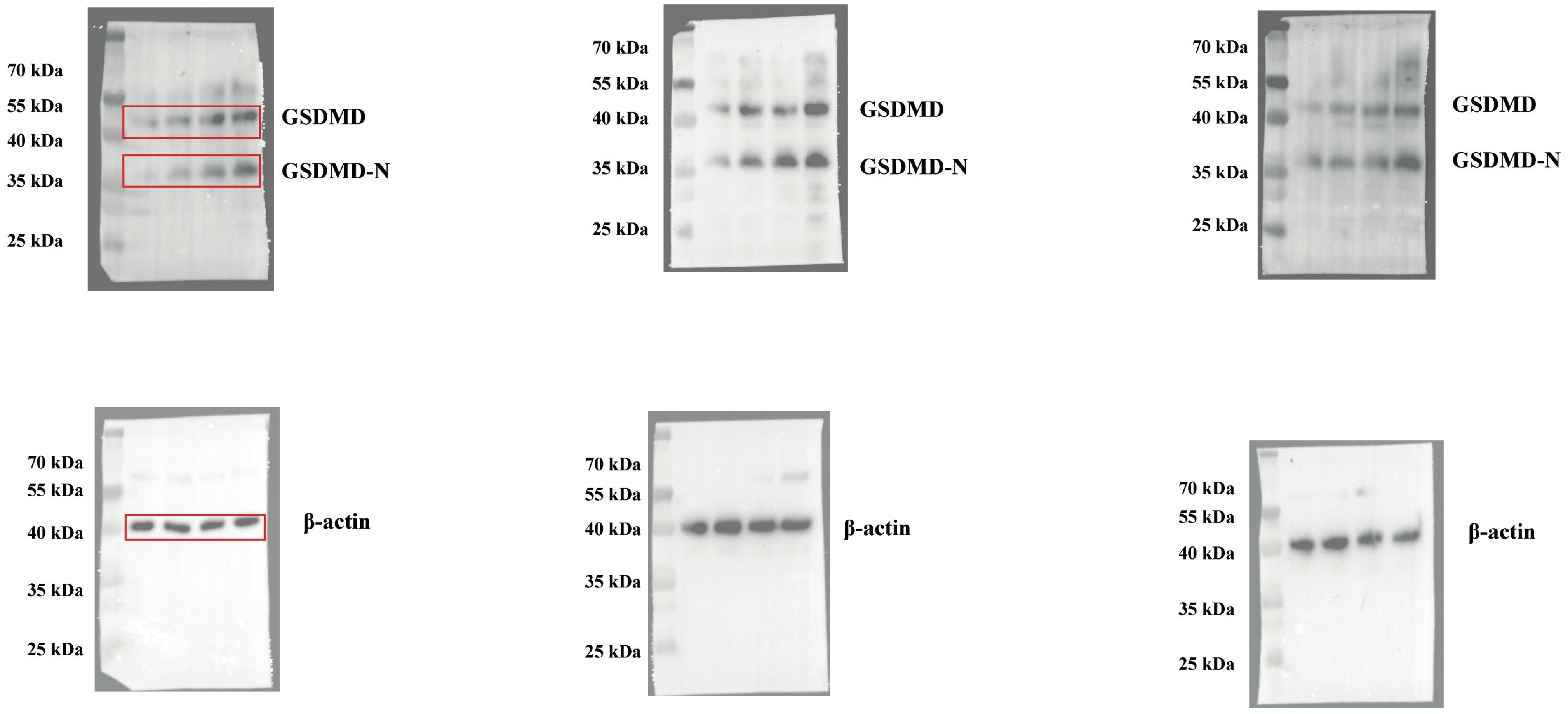

Fig 4. A

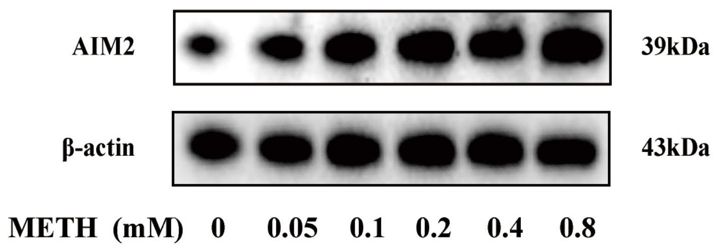

Full unedited gels for Fig 4. A

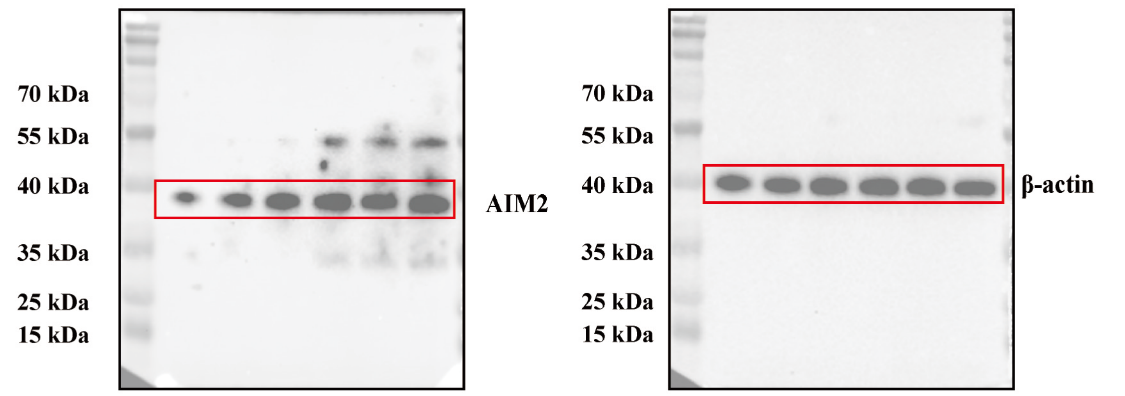

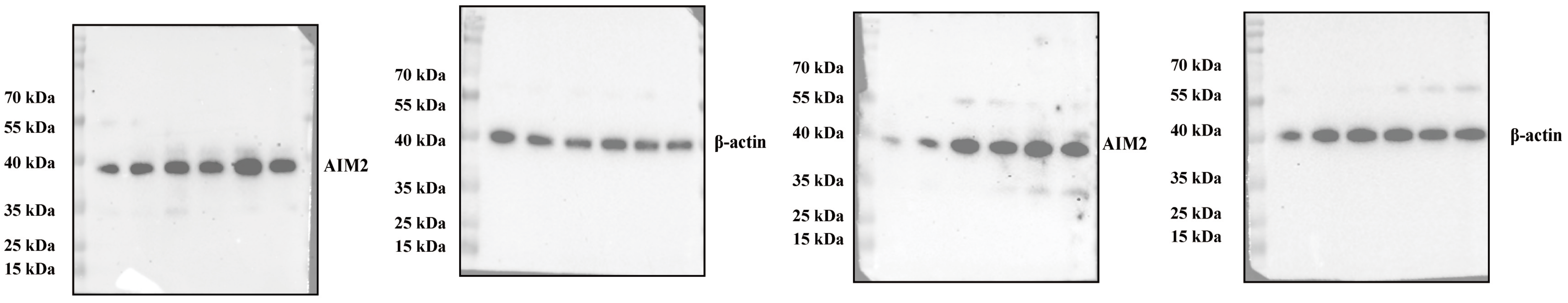

Fig 4. B

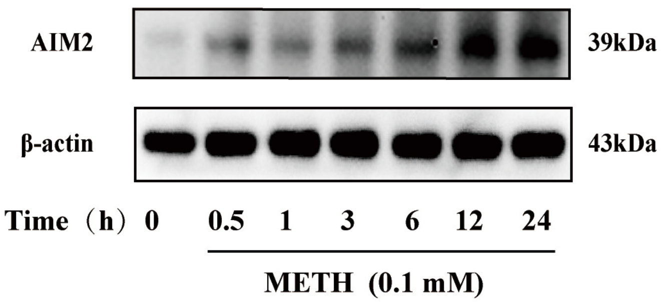

Full unedited gels for Fig 4. B

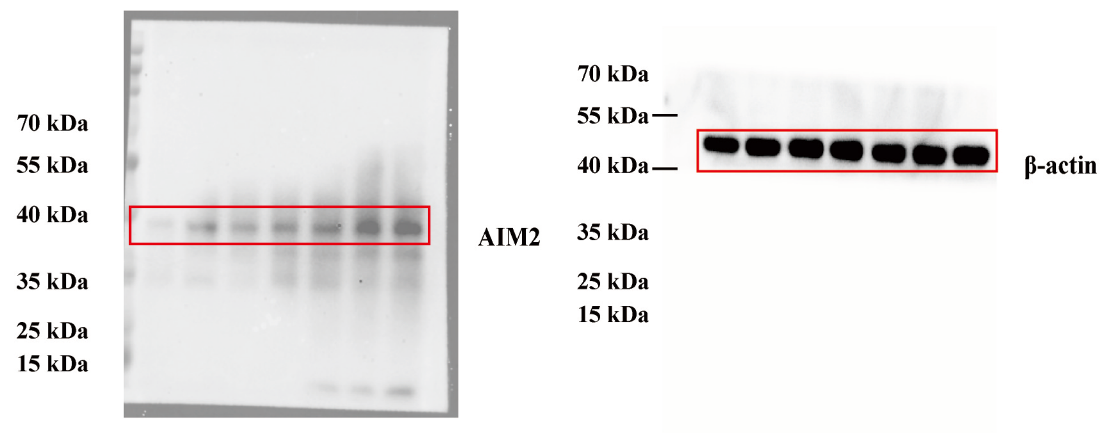

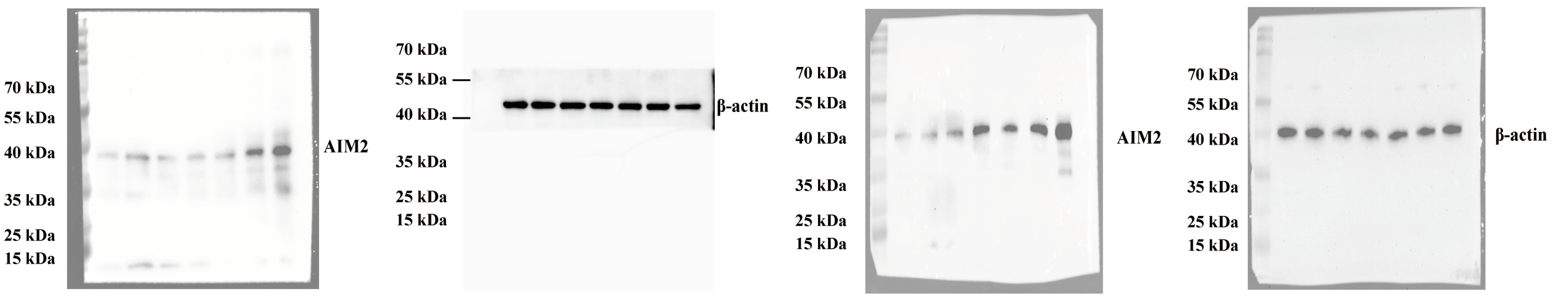

Fig 4. C

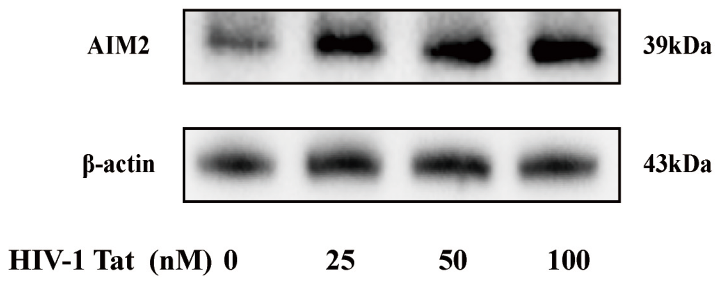

Full unedited gels for Fig 4. C

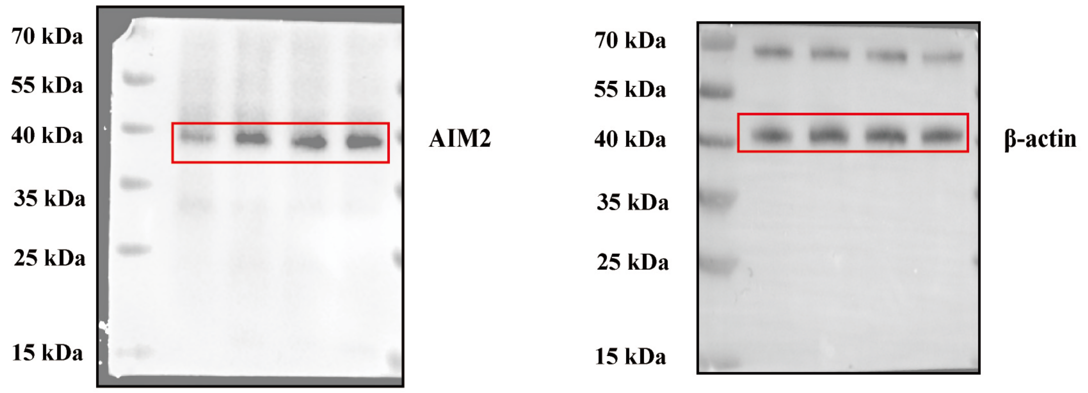

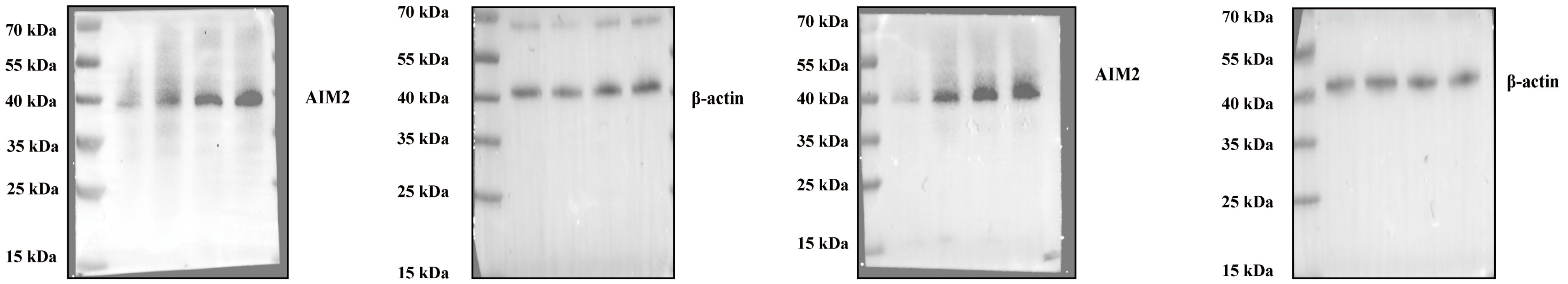

Fig 4. D

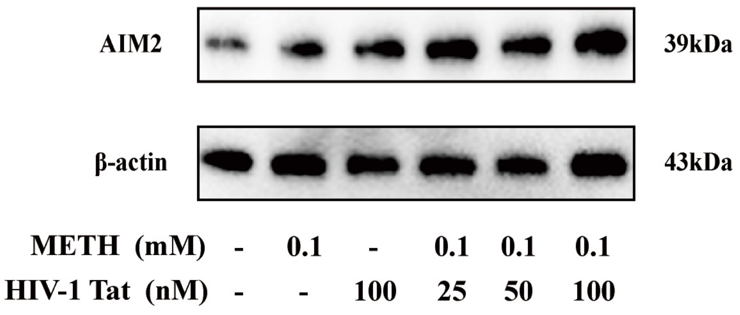

Full unedited gels for Fig 4. D

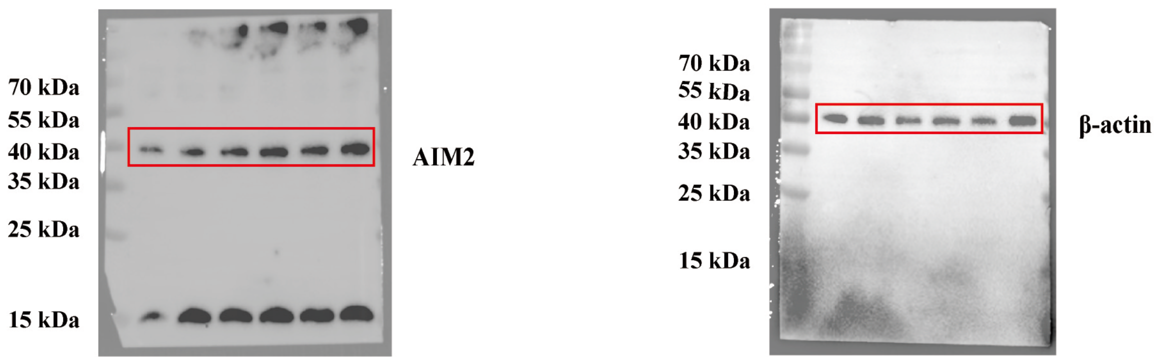

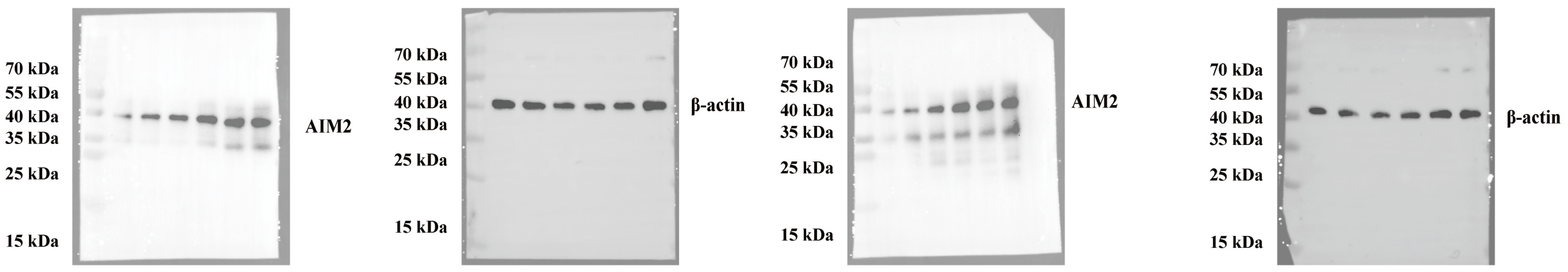

Fig 5.B

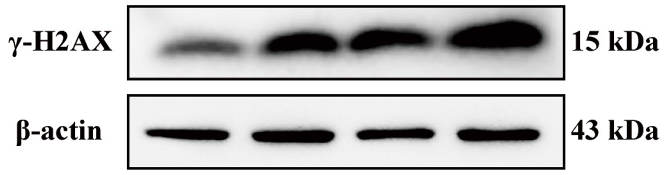

Full unedited gels for Fig 5. B

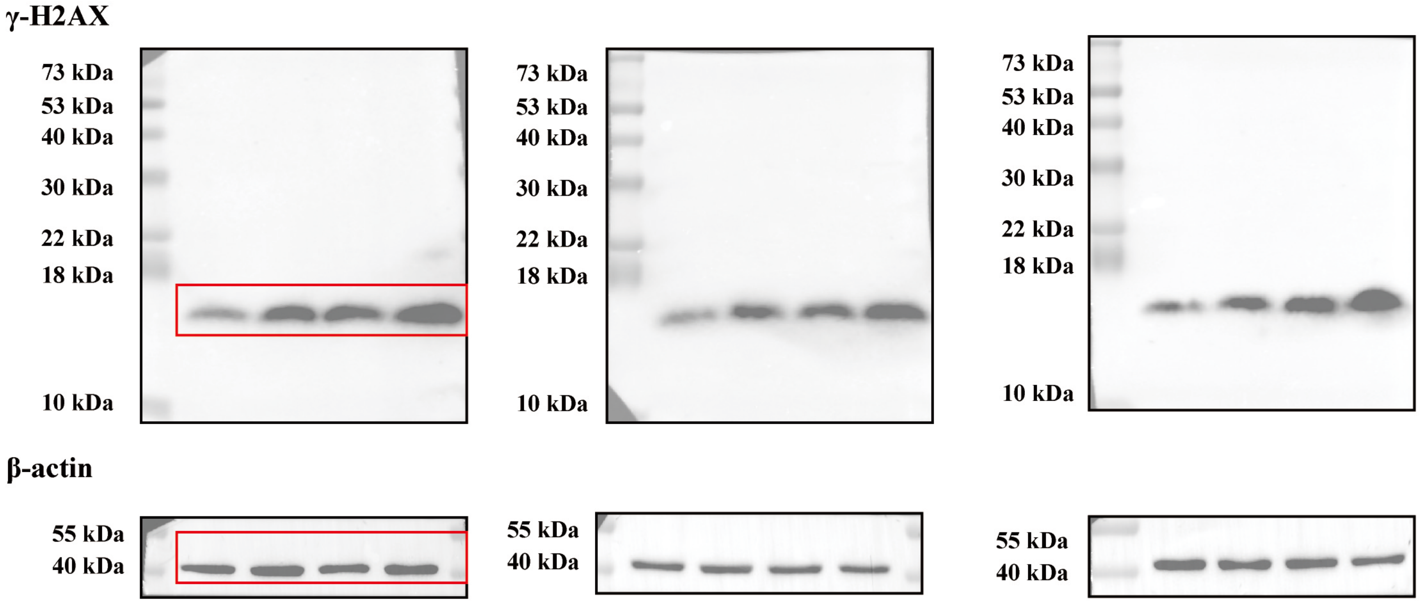

Fig 5.C

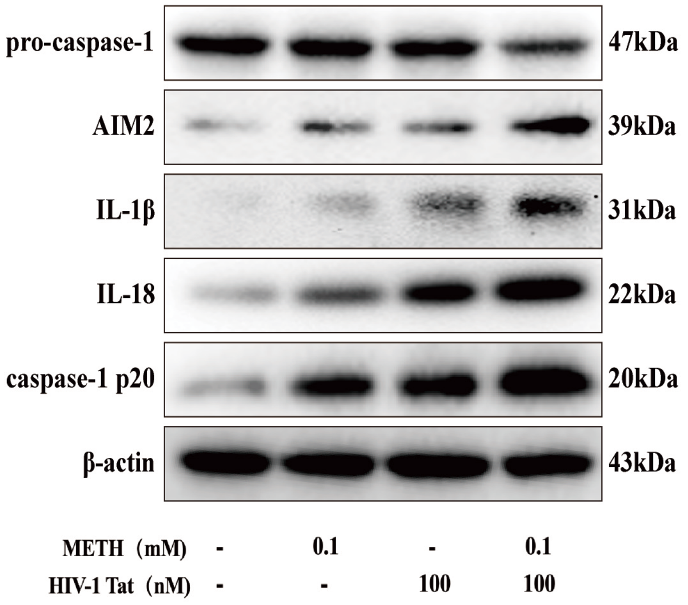

Full unedited gels for Fig 5. C

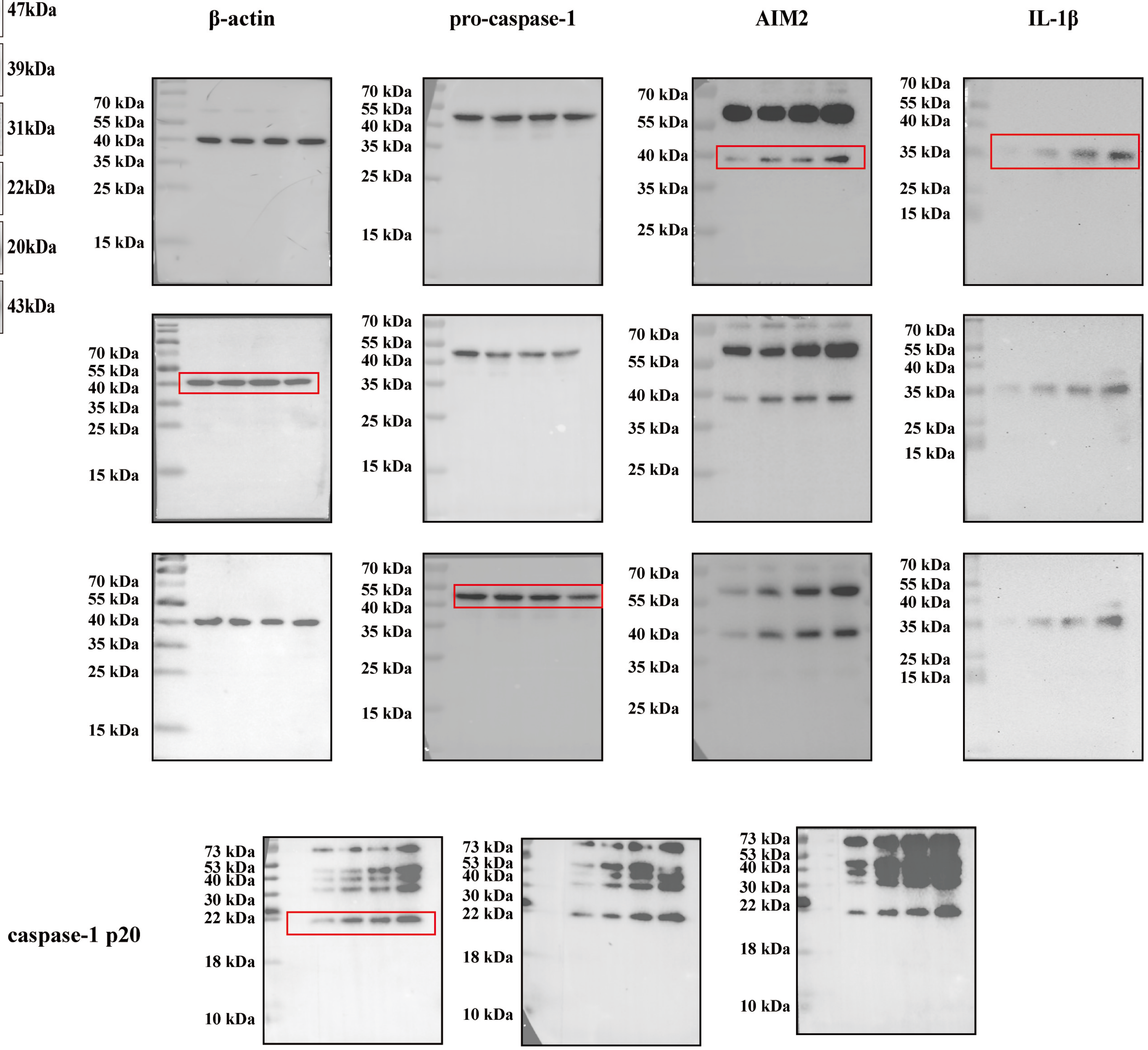

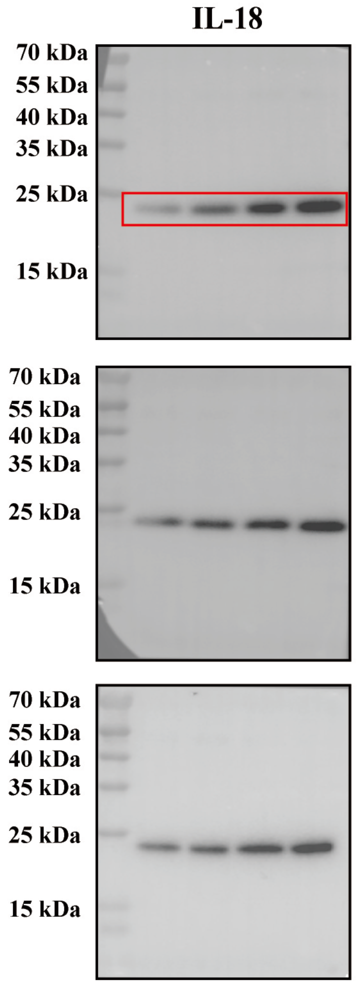

Supplementary Fig. 1 B

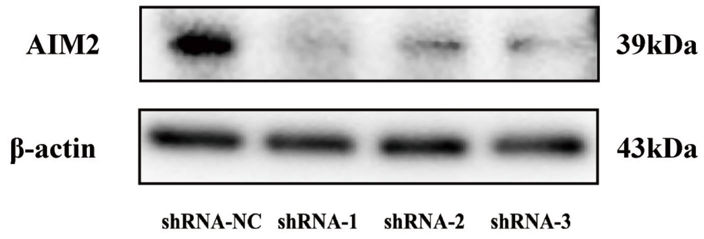

Full unedited gels for Supplementary Fig. 1 B

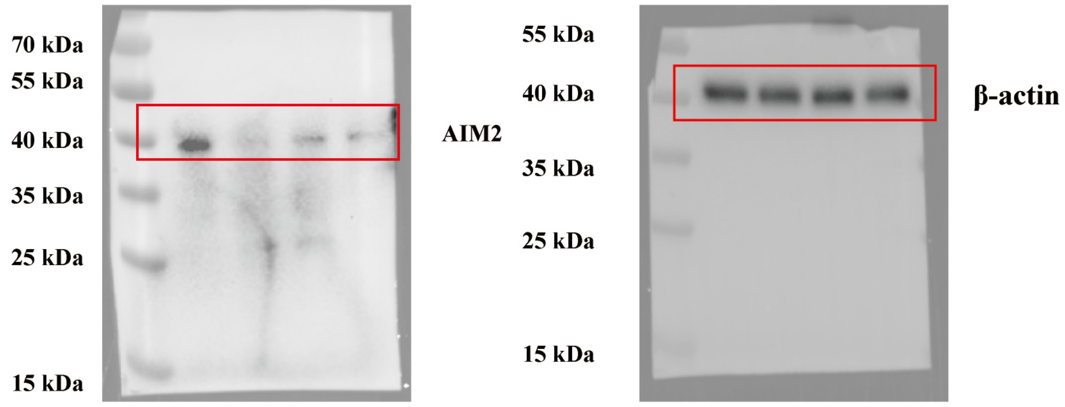

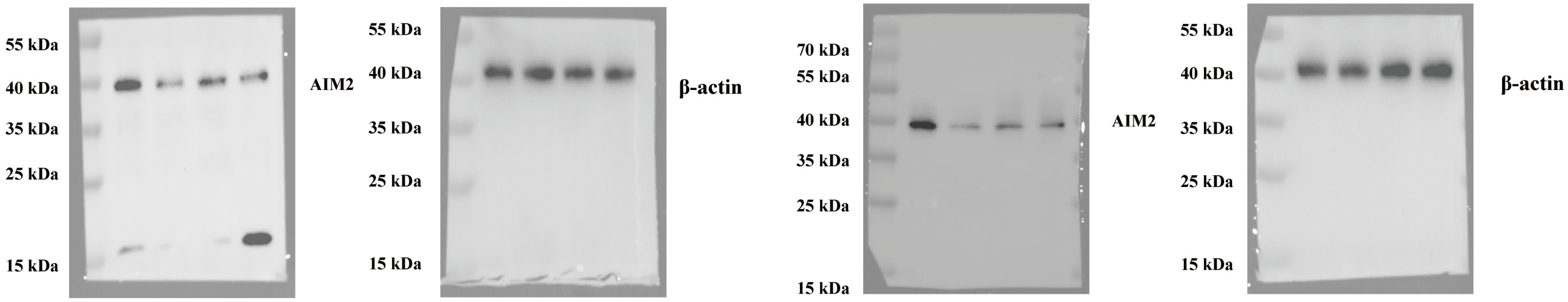

Supplementary Fig. 1 E

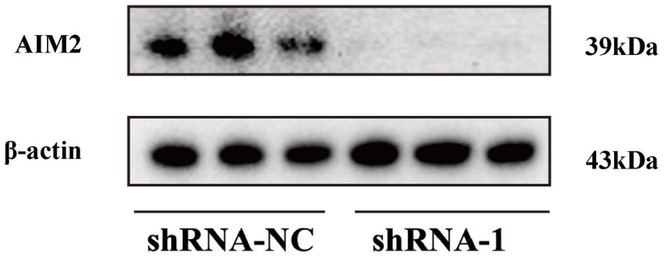

Full unedited gels for Supplementary Fig. 1 E

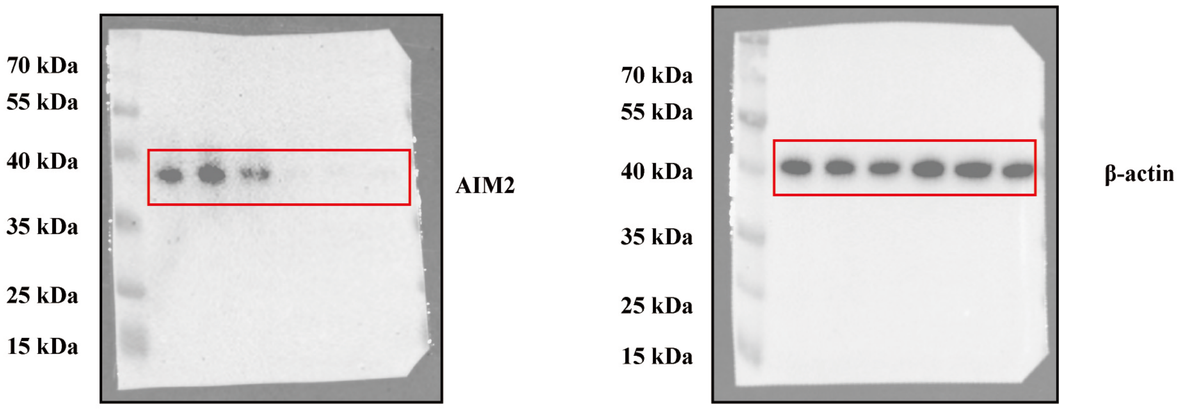

Fig 6.A

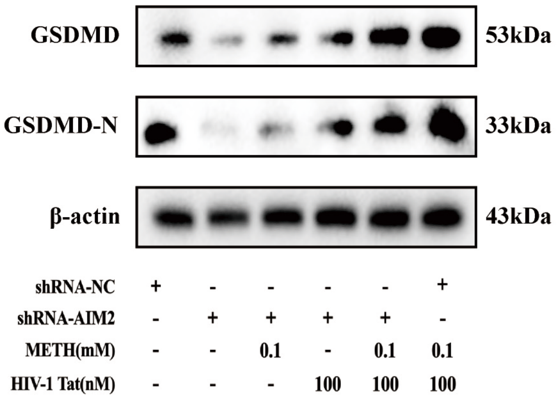

Full unedited gels for Fig 6. A

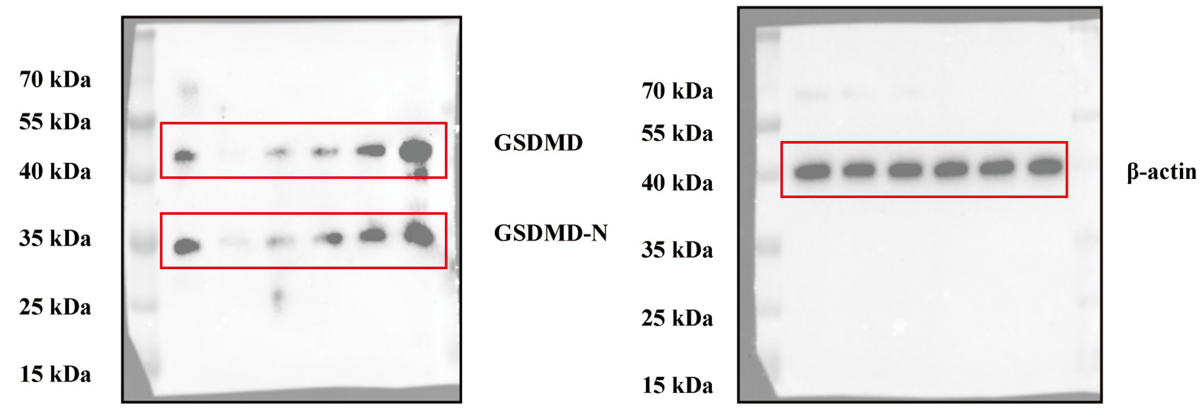

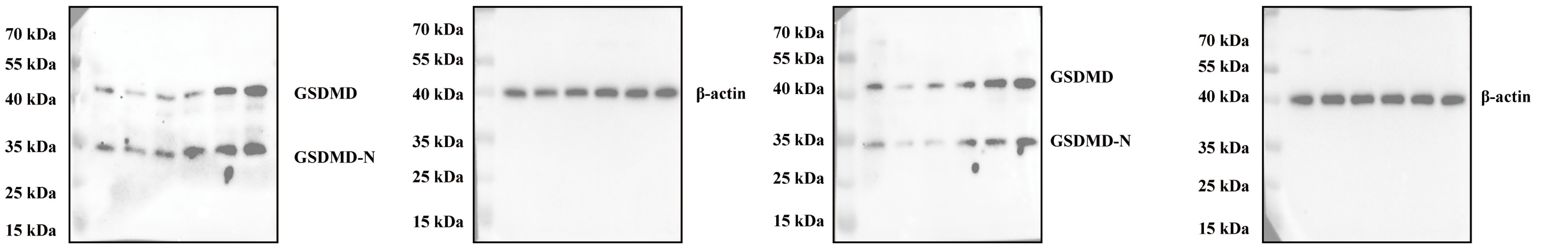

Fig 6.B

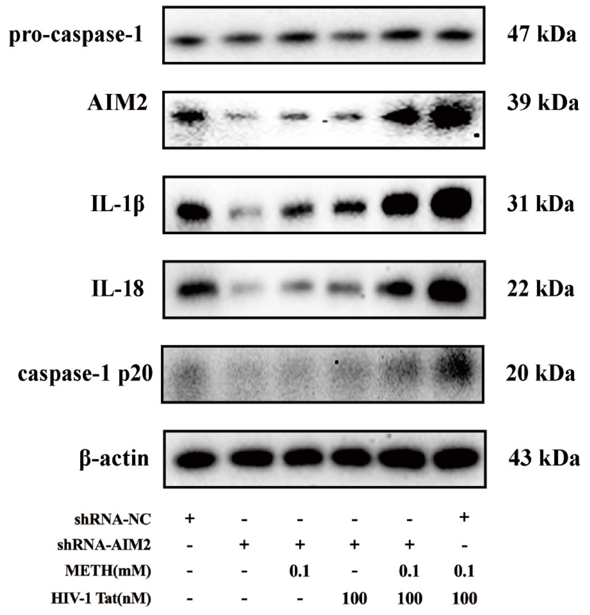

Full unedited gels for Fig 6. B

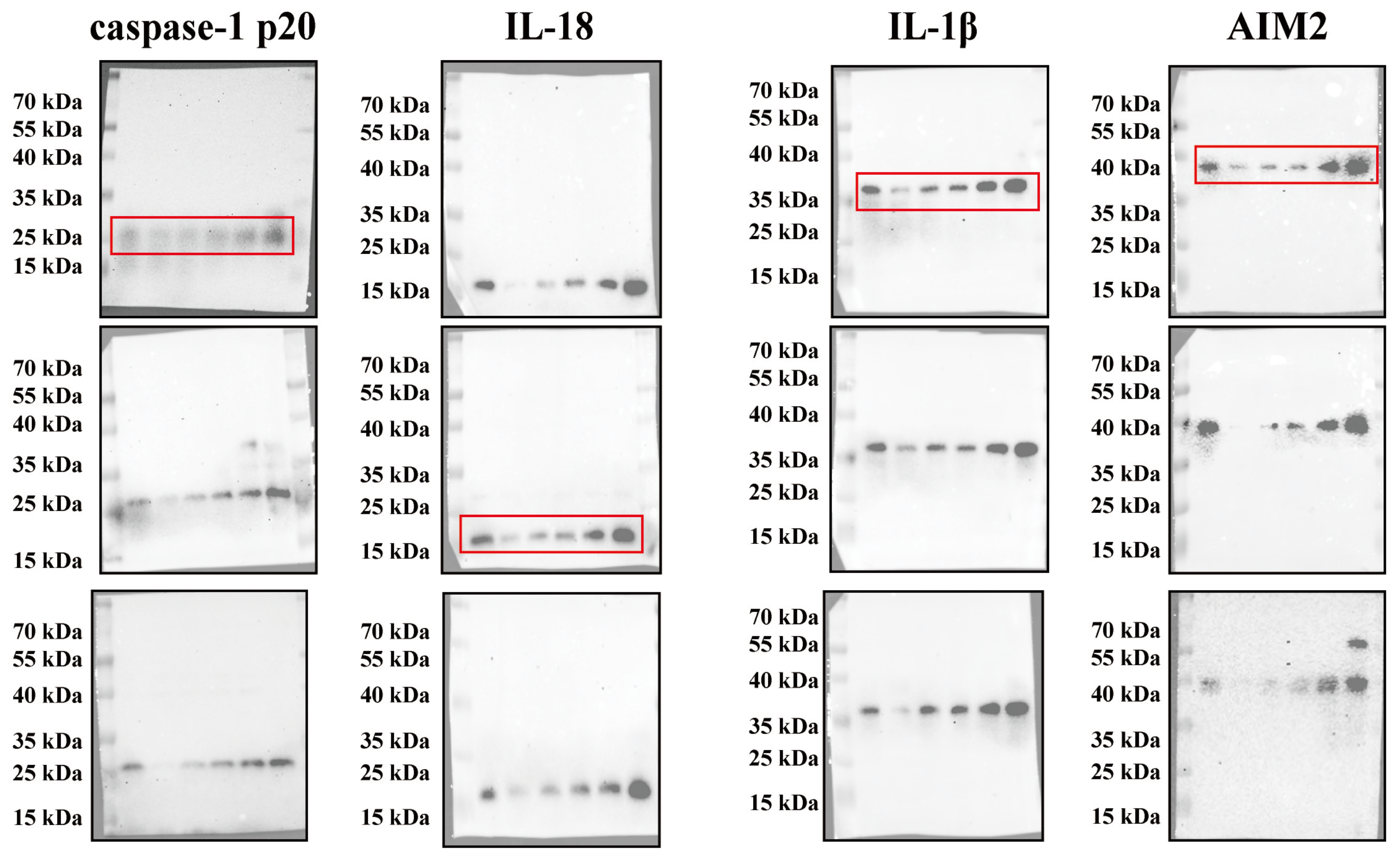

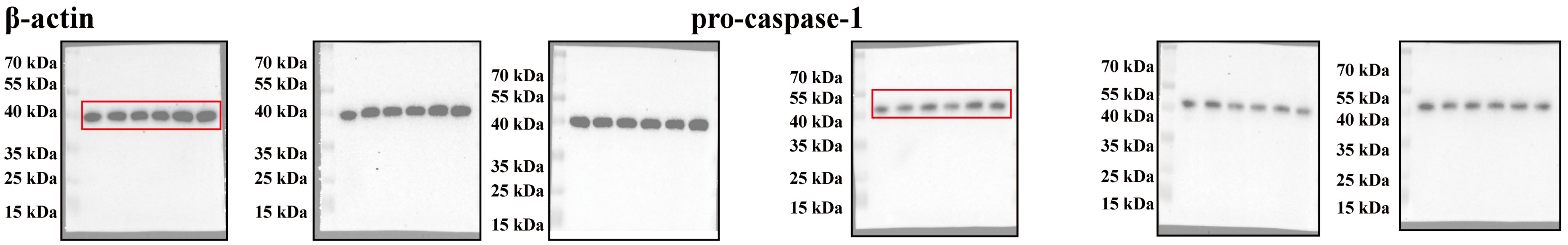

Supplement: Supplementary file 3 — Supplementary file3 (PDF 27689 KB) [file 10753_2025_2266_MOESM3_ESM.pdf]
